# Evaluation of anifrolumab safety in systemic lupus erythematosus: A meta-analysis and systematic review

**DOI:** 10.3389/fimmu.2022.996662

**Published:** 2022-09-23

**Authors:** Zhihui Liu, Ruijuan Cheng, Yi Liu

**Affiliations:** ^1^Department of Rheumatology and Immunology, West China Hospital, Sichuan University, Chengdu, China; ^2^Rare Diseases Center, West China Hospital, Sichuan University, Chengdu, China; ^3^Institute of Immunology and Inflammation, Frontiers Science Center for Disease-Related Molecular Network, West China Hospital, Sichuan University, Chengdu, China

**Keywords:** systemic lupus erythematosus, type I interferon, anifrolumab, RCTs, meta-analysis

## Abstract

**Objectives:**

Systemic lupus erythematosus (SLE) is a chronic autoimmune disease, and type I interferon plays an important role in its pathogenesis. Anifrolumab is a new strategy for the treatment of systemic lupus erythematosus. It could antagonize the activity of all type 1 interferons by binding with type I interferon receptor subunit 1. The aim of our study was to evaluate the safety of anifrolumab in patients with moderate to severe SLE (excluding patients with active severe lupus nephritis or central nervous system lupus).

**Methods:**

Four databases (Embase, Cochrane, PubMed, Web of Science) were systematically searched from inception until December 2021 for randomized controlled trials (RCTs) evaluating the safety of anifrolumab versus placebo in SLE patients. Then, the incidence of adverse events in each study was aggregated using meta-analysis.

**Results:**

A total of 1160 SLE patients from four RCTs were included in the analysis. Serious adverse events were less common in the anifrolumab group than in the placebo group (RR: 0.76, 95% CI: 0.59-0.98, p<0.03). The most common adverse events included upper respiratory tract infection (RR: 1.48, 95% CI: 1.13-1.94, P=0.004), nasopharyngitis (RR: 1.66, 95% CI: 1.25-2.20, P=0.0004), bronchitis (RR: 1.96, 95% CI: 1.32-2.92, P=0.0009), and herpes zoster (RR: 3.40, 95% CI: 1.90-6.07, P<0.0001).

**Conclusion:**

Anifrolumab is considered a well-tolerated option for the treatment of SLE patients with good safety.

**Systematic Review Registration:**

https://inplasy.com, identifier 202230054.

## Introduction

Systemic lupus erythematosus (SLE) is a chronic, autoimmune, systemic disease involving multiple organs. SLE predominantly occurs in women of childbearing age and is also one of the main causes of death. The incidence and prevalence of SLE in North America were 3.7 to 49 per 100,000 person-years and 48 to 366.6 per 100,000 individuals, respectively; the annual incidence of SLE in Asia was 2.8 to 8.6/100,000 person-years, and the prevalence was 26.5 to 103/100,000 person-years (1). The pathogenesis of SLE is complex and includes immune system dysregulation, genetic susceptibility, environmental inducement, innate and adaptive immune system activation and other factors (2).

The traditional drug options for SLE include glucocorticoids and immunosuppressants, but these drugs may cause an increased risk of many adverse reactions, such as infection and mortality. The development of targeted biological agents to control disease is a priority. However, the results of current B-cell-targeting biologics (including rituximab, belimumab, etc.) are not satisfactory, and only belimumab has been approved by the Food and Drug Administration (FDA) and China Food and Drug Administration (CFDA) for SLE (3). Type I interferons have an important role in the pathogenesis of SLE, and a signature of high type I interferon expression is present in more than 60% of patients with SLE. The type I interferon gene expression signature is significantly positively correlated with the disease activity index (SLEDAI) score of SLE patients (4–6). Anifrolumab is a fully human IgG1k monoclonal antibody that binds to subunit 1 of the type I interferon receptor (IFNAR), thereby antagonizing all type 1 interferon-related activities (5).

At present, several randomized controlled trials (RCTs) have investigated the effects of different doses of anifrolumab on SLE. These clinical studies have shown that in moderate-to-severe SLE, administration of intravenous anifrolumab every 4 weeks significantly improves disease activity at multiple clinical endpoints compared with placebo (7–9). The result of a phase II RCT showed that subcutaneous anifrolumab every 2 weeks in patients with SLE is consistent with previous studies of intravenous anifrolumab (10). However, some adverse events (AEs) may also occur during the use of anifrolumab, such as herpes zoster, infection events, headache, diarrhea and so on. A previous meta-analysis of 3 RCTs showed that intravenous injection of 300 mg of anifrolumab was significantly effective in patients with SLE, but this analysis did not report in detail the adverse events of anifrolumab (11). In this analysis, adverse events, such as serious adverse events, adverse events leading to discontinuation, malignancy, tuberculosis, infusion-related reaction and infection, were not detailed described in the patients receiving anifrolumab. Therefore, our study tried to compare and evaluate the safety of anifrolumab subgroups versus placebo as treatment for patients with active SLE by performing a systematic review and meta-analysis of RCTs.

## Methods

### Study design and research

We searched published RCTs to evaluate the safety of anifrolumab. The search period was from inception to December 30, 2021, and the data were obtained from four databases (PubMed, Cochrane, Web of Science, and EMBASE). The retrieval strategy was the combination of subject words and free words, as follows: ((“Lupus Erythematosus, Systemic”[Mesh]) OR (((((Systemic Lupus Erythematosus[Title/Abstract]) OR (Lupus Erythematosus Disseminatus[Title/Abstract])) OR (Libman-Sacks Disease[Title/Abstract])) OR (Disease, Libman-Sacks[Title/Abstract])) OR (Libman Sacks Disease[Title/Abstract]))) AND ((“anifrolumab” [Supplementary Concept]) OR (((MEDI-546[Title/Abstract])) OR (Anifrolumab[Title/Abstract]))).

The study was performed in accordance with the preferred reporting system for systematic reviews (PRISMA), and registered with the International Platform of Registered Systematic Review and Meta-analysis Protocols (INPLASY202230054).

### Study selection

Studies were performed independently by two investigators, and study characteristics were extracted. The study would be included in the final analysis if it met all the following criteria: 1) the study was an RCT comparing the efficiency of anifrolumab with placebo in the treatment of active SLE. And 2) noncomparative, nonrandomized (such as case–control, cohort or cross-sectional) and were not written in English were excluded; 3) the original articles that could not provide valid data, were incomplete or had incomplete final data were excluded; 4) animal experiments were excluded; 5) systematic reviews, guidelines, conference abstracts, or experience reports were excluded.

### Data extraction

We used the literature management software (EndNoteX9) to merge all the obtained documents. First, after eliminating duplicate documents, a brief title/summary review was used to conduct a preliminary search of the retrieved literatures. Next, we select the articles through the review of the full text articles to decide whether to include the research in the final analysis. Finally, we collected the study characteristics (such as the first author, year of publication, study design and trial stage), patient characteristics (such as the number of cases, the number of control groups, dosage, frequency of administration) and clinical results (number of adverse reactions). This study required neither institutional review committee approval nor patient informed consent.

### Assessment risk of bias

The bias of the included studies was systematically evaluated using the Cochrane ROB 2 tool (12). The items used to evaluate each research include: adequacy of sequence generation, blinding of subjects and personnel, blinding of outcome evaluation, concealment of allocation, selective outcome reporting, dropout processing (incomplete outcome data), and other potential sources of bias. The risk of bias assessment was performed independently by 3 reviewers. Disagreements were resolved by consensus.

### Statistical analysis

The Review Manager (Revman) version 5.4 software was selected for statistical analysis (Cochrane Collaboration nordice Cochrane Center, Copenhagen, Denmark, 2020). P<0.05 was considered significant. For dichotomous data, relative risk (RR) was used as the effect statistic, and the 95% confidence interval (CI) was used for interval estimation. The χ2 test was used to judge the statistical heterogeneity. If there was no statistical heterogeneity (P>0.10, I2 ≤50%), we used the fixed effect model. Otherwise, we selected the random-effects model. Subgroup analyses were performed according to adverse events.

### Patient and public involvement

No patients are involved.

## Results

### Study search and selection

**Figure 1:**
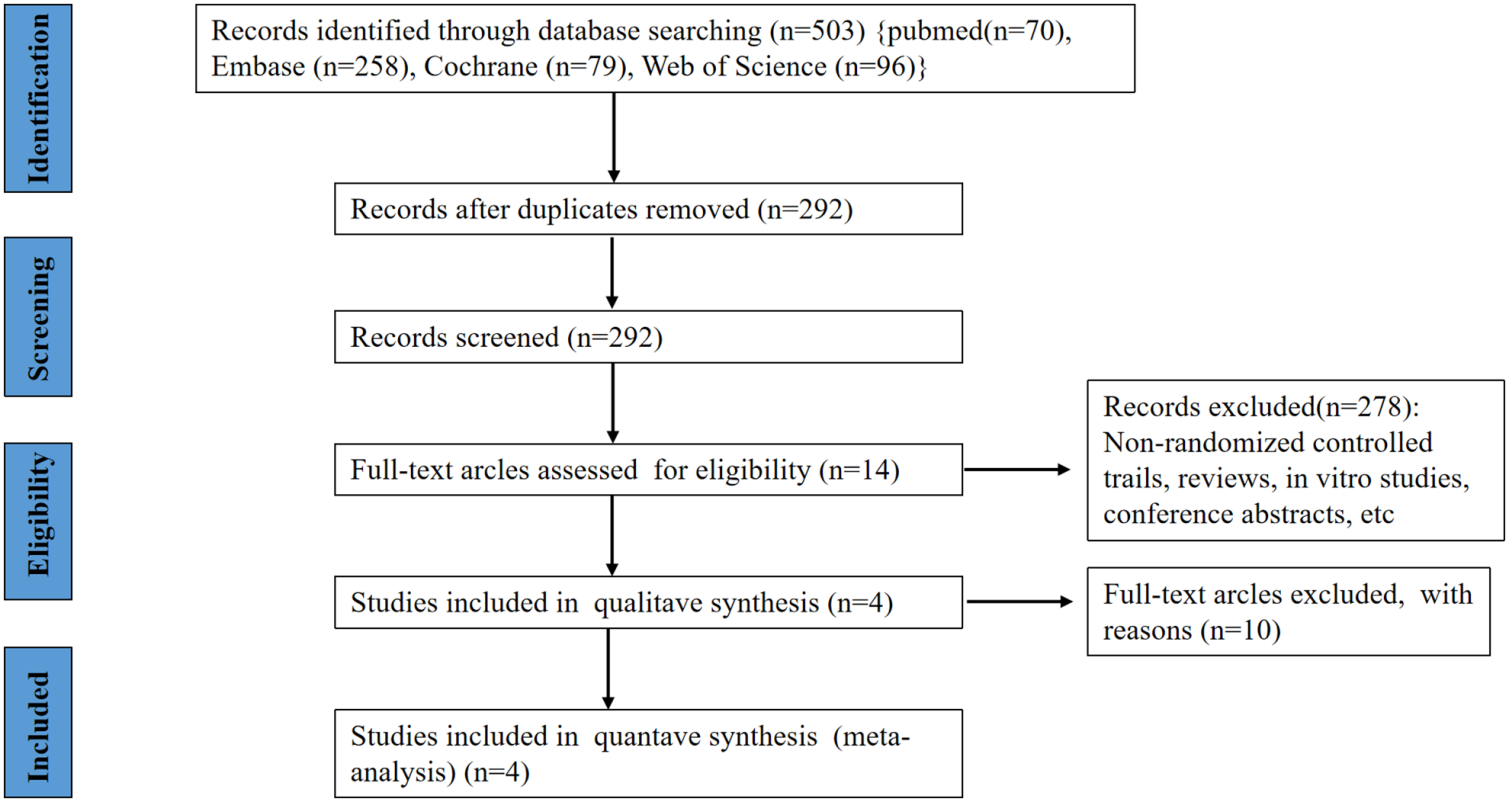
Flow chart of search strategy and study selection process.

**Figure 1** shows how our research choices were made. A total of 503 articles were identified through a preliminary search of relevant databases from database establishment to December 31, 2021 (70 records were found on PubMed, 79 additional records were found on Cochrane, 96 records were found on Web of Science and 258 records were found on EMBASE). After the initial screening, 14 studies and trial results were obtained. After reading the full text and rescreening, 4 RCTs were finally obtained, including a total of 1160 patients, of which 684 patients were assigned to receive anifrolumab and 476 were assigned to receive placebo (7–10). The RCTs included in our study were all in English.

### Basic characteristics of the included studies

**Figure 2:**
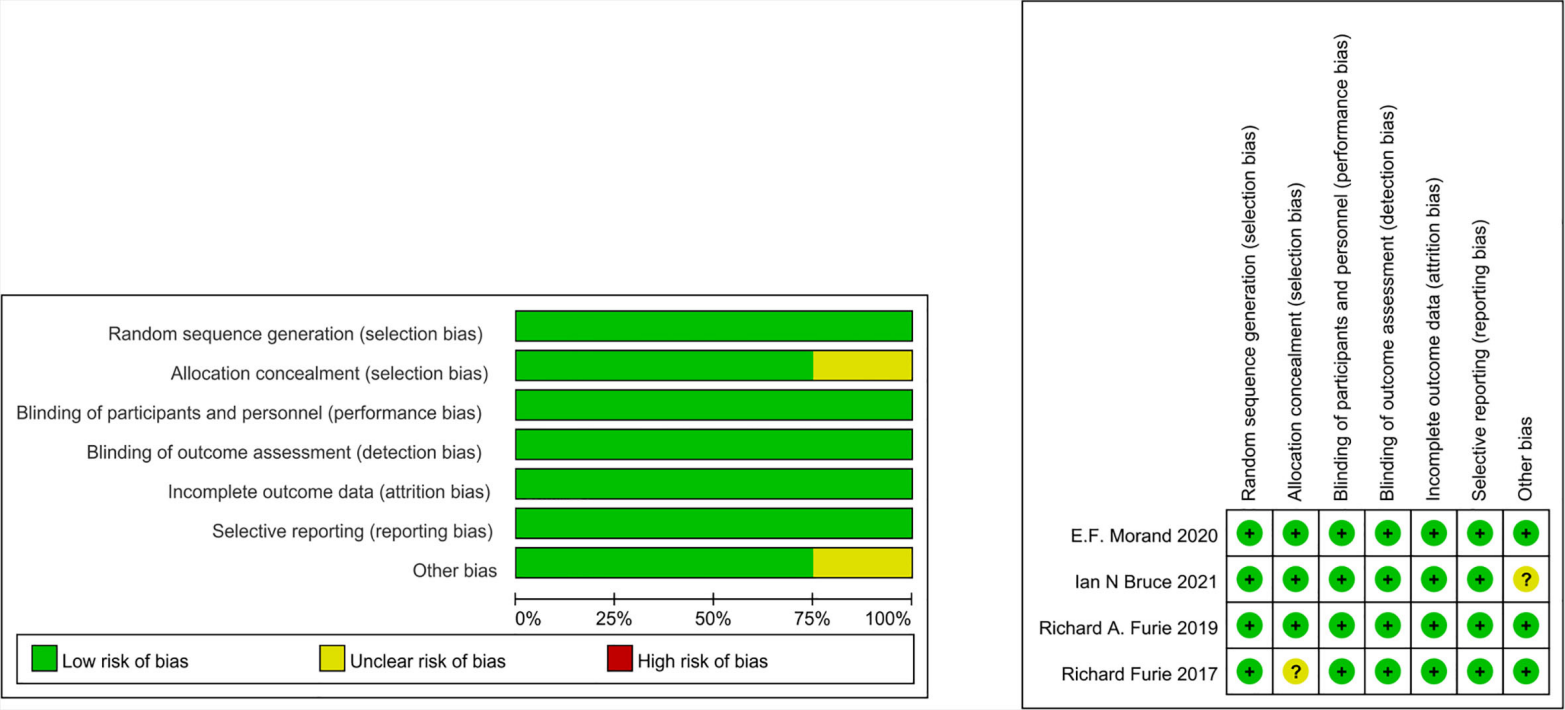
Summary of risk of bias of included studies.

**Table 1** summarizes the baseline characteristics. The low risk of bias represents that these studies reflect true treatment and adverse effects. The findings are likely to be reliable, as there are no primary or secondary sources of bias that could have influenced our results, supporting our conclusions with evidence (**Figure 2**).

**Table 1:** Characteristics of the studies included in the meta-analysis.

	Furie R, 2017.(NCT01438489)			Furie R, 2019.(NCT02446912)			Morand EF, 2020.(NCT02446899)		Bruce I. N. 2021.(NCT02962960)		
	Placebo	Anifrolumab300mg	Anifrolumab1000mg	Placebo	Anifrolumab150mg	Anifrolumab300mg	Placebo	Anifrolumab300mg	Placebo	Anifrolumab150mg	Anifrolumab300mg
Number of patients	101	99	105	184	93	180	182	180	9	14	13
Age, years, mean (SD)	39.3 (12.9)	39.1 (11.9)	40.8 (11.6)	41.0 (12.30)	40.8 (12.05)	42.0 (11.99)	41.1 (11.5)	43.1 (12.0)	47.8 (14.2)	46.3 (9.1)	41.5 (9.2)
Female, n, (%)	93 (91.2)	93 (93.9)	99 (95.2)	171 (92.9)	86 (92.5)	165 (91.7)	170 (93.4)	168 (93.3)	8 (89%)	12 (86%)	12 (92%)
Trial cycle/week	52	52	52	52	52	52	52	52	52	52	52
Serious adverse events, (%)	19 (18.8)	16 (16.2)	18 (17.1)	30 (16.3)	10 (10.8)	25 (13.9)	31 (17.0)	15 (8.3)	0	4 (29%)	2 (15%)
Death, (%)	0 (0.0)	0 (0.0)	1 (1.0)	0	0	1 (0.6)	0	1 (0.6)	0	0	0
Adverse events leading to discontinuation, (%)	8 (7.9)	3 (3.0)	10 (9.5)	5 (2.7)	5 (5.4)	11 (6.1)	13 (7.1)	5 (2.8)	0	2(14)	3 (23)
Headache, (%)	13 (12.9)	12 (12.1)	12 (11.4)	16 (8.7)	6 (6.5)	17 (9.4)	N	N	0	2 (14)	2 (15)
Upper respiratory tract infection, (%)	10 (9.9)	13 (13.1)	11 (10.5)	18 (9.8)	16 (17.2)	22 (12.2)	18 (9.9)	39 (21.7)	3 (33)	5 (36)	1 (8)
Nasopharyngitis, (%)	4 (4.0)	12 (12.1)	12 (11.4)	22 (12.0)	14 (15.1)	36 (20.0)	20 (11.0)	28 (15.6)	1 (11)	3 (21)	2 (15)
Urinary tract infection, (%)	11 (10.9)	15 (15.2)	7 (6.7)	27 (14.7)	9 (9.7)	22 (12.2)	25 (13.7)	20 (11.1)	N	N	N
Bronchitis, (%)	4 (4.0)	7 (7.1)	9 (8.6)	10 (5.4)	7 (7.5)	16 (8.9)	7 (3.8)	22 (11.1)	1 (11)	1 (7)	3 (23)
Herpes zoster, (%)	2 (2.0)	5 (5.1)	10 (9.5)	3 (1.6)	5 (5.4)	10 (5.6)	2 (1.1)	13 (7.2)	1 (11)	3 (21)	0
Influenza, (%)	2 (2.0)	6 (6.1)	8 (7.6)	2 (1.1)	1 (1.1)	2 (1.1)	6 (3.3)	4 (2.2)	0	1 (7)	0
Diarrhea, (%)	4 (4.0)	4 (4.0)	8 (7.6)	N	N	N	N	N	N	N	N
Sinusitis, (%)	3 (3.0)	6 (6.1)	6 (5.7)	N	N	N	9 (4.9)	12 (6.7)	N	N	N
Cough, (%)	2 (2.0)	3 (3.0)	8 (7.6)	N	N	N	6 (3.3)	10 (5.6)	N	N	N
Malignancy, (%)	N	N	N	1 (0.5)	1 (1.1)	3 (1.7)	1 (0.5)	0	0	0	0
Non-opportunistic infections, (%)	N	N	N	8 (4.3)	2 (2.2)	9 (5.0)	10 (5.5)	5 (2.8)	0	0	0
Opportunistic infections, (%)	N	N	N	1 (0.5)	0	1 (0.6)	N	N	0	0	0
Infusion-related reaction, (%)	6	2	4	13 (7.1)	9 (9.7)	16 (8.9)	14 (7.7)	25 (13.9)	N	N	N
Anaphylaxis, (%)	N	N	N	0	1 (1.1)	0	N	N	0	0	0
Gastroenteritis, (%)	N	N	N	N	N	N	0	2 (1.1)	0	0	0
Tuberculosis, (%)	N	N	N	1 (0.5)	0	1 (0.6)	0	3 (1.7)	0	0	1 (8)

Of the 4 RCTs, 3 were all intravenous anifrolumab or placebo administered every 4 weeks for 52 weeks (NCT01438489, NCT02446912, NCT02446899) (7–9), and 1 was subcutaneous anifrolumab or placebo every 2 weeks for 52 weeks (NCT02962960) (10). In the MUSE (NCT01438489) phase II trial, 305 patients with active SLE between the ages of 18 and 65 were randomized to an intervention dose of anifrolumab 300 mg/4 weeks (n=99), anifrolumab 1000 mg/4 weeks (n=105), and placebo (n=101). A total of 457 patients with active SLE between the ages of 18 and 70 were included in the TULIP-1 (NCT02446912) study and were randomly assigned to 3 groups: anifrolumab 150 mg (n=93), anifrolumab 300 mg (n=180) and placebo (n=184). The TULIP-2 (NCT02446899) trial included 362 patients randomized to anifrolumab 300 mg (n=180) or placebo (n=182). The NCT02962960 trial included 36 patients randomized to receive anifrolumab 150 mg (n=14), anifrolumab 300 mg (n=13), and placebo (n=9) subcutaneously every 2 weeks.

### Meta-analysis of adverse effects

Adverse events were documented in 4/4 of the studies, and the results of our meta-analysis are summarized in **Supplementary Figure 1** and **Table 2**.

**Table 2:** Adverse events.

adverse events	Anifrolumab(N)	placebo(N)	RR	95%CI	*P value*	Study heterogeneity			
						Chi2	df	I^2^, %	*P value*
serious adverse events	90 (13.16%)	80 (16.81%)	0.78	0.60-1.00	0.05	6.27	6	4	0.39
death	3 (0.44%)	0	2.99	0.47-18.91	0.24	0.00	2	0	1.00
Adverse events leading to discontinuation	39 (5.70%)	26 (5.46%)	1.05	0.69-1.62	0.81	10.99	6	45	0.09
headache	51 (10.12%)	29 (9.86%)	0.99	0.69-1.42	0.94	1.97	5	0	0.85
Upper respiratory tract infection	107(15.64%)	49 (10.29%)	1.48	1.13-1.94	0.004	6.85	6	12	0.34
Nasopharyngitis	107(15.64%)	47 (9.87%)	1.66	1.25-2.20	0.0004	3.31	6	0	0.77
Urinary tract infection	73 (11.11%)	63 (13.49%)	0.83	0.62-1.11	0.21	2.78	4	0	0.60
Bronchitis	65 (9.50%)	22 (4.62%)	1.96	1.32-2.92	0.0009	2.81	6	0	0.83
Herpes zoster	46 (6.73%)	8 (1.68%)	3.40	1.90-6.07	<0.0001	4.21	6	0	0.65
Influenza	22 (3.22%)	10 (2.10%)	1.62	0.83-3.13	0.15	4.15	5	0	0.53
Sinusitis	24 (6.25%)	12 (4.24%)	1.60	0.85-3.01	0.14	0.35	2	0	0.84
Cough	21 (5.47%)	8 (2.83%)	2.10	1.00-4.40	0.050	0.92	2	0	0.630
Tuberculosis	5 (1.04%)	1 (0.27%)	2.09	0.54-8.16	0.29	1.42	3	0	0.70
Malignancy	4 (0.83%)	2 (0.53%)	1.54	0.38-6.20	0.54	1.26	2	0	0.53
Infusion-related reaction	56 (8.52%)	33 (7.07%)	1.24	0.87-1.79	0.24	5.14	4	22	0.27
Anaphylaxis	1 (0.33%)	0	5.9	0.24-143.54	0.28	NA	NA	NA	NA
Non-opportunistic infections	16 (3.33%)	18 (4.80%)	0.72	0.39-1.35	0.31	1.64	2	0	0.44
Opportunistic infections	1 (0.33%)	1 (0.52%)	0.84	0.10-6.69	0.87	0.04	1	0	0.84

NA, Not applicable.

### Serious adverse events

The meta-analysis showed that the incidence of serious adverse events was significantly lower in the anifrolumab group than in the placebo group [relative risk (RR): 0.78, 95% confidence interval (95% CI): 0.60-1.00, P=0.05]. In the subgroup analysis, there were no significant differences among the 150 mg subgroup (RR: 0.81, 95% CI: 0.44-1.52, P=0.52), 1000 mg subgroup (RR: 0.91, 95% CI: 0.51-1.63, P=0.76) and placebo group, while the 300 mg subgroup (RR: 0.73, 95%: 0.54-1.00, P=0.05) had a lower incidence than the placebo group. Deaths in patients receiving anifrolumab were rare, with 3 deaths among 684 patients receiving anifrolumab and none among those receiving placebo (RR: 2.99, 95% confidence interval: 0.47 -18.91, P=0.24). All of these 3 patients died after receiving anifrolumab. Two of the deaths were due to pneumonia and one was due to acute colitis. The incidence of adverse events leading to intervention discontinuation was not significantly different between the two groups. (RR: 1.05, 95% CI: 0.69-1.62, P=0.81).

### Respiratory tract infection

Compared with placebo, patients receiving anifrolumab were more likely to develop respiratory tract infections. The incidence of upper respiratory tract infection in the overall anifrolumab group was significantly higher than that in the placebo group (RR: 1.48, 95% CI: 1.13-1.94, P = 0.004), while there were similar changes in the 300 mg subgroup (RR: 1.53, 95% CI: 1.10-2.15, P = 0.01). However, no significant difference was found between the treatment group and the placebo group in the 150 mg subgroup (RR: 1.60, 95% CI: 0.92-2.77, P = 0.09) and 1000 mg subgroup (RR: 1.06, 95% CI: 0.47-2.38, P = 0.89). The incidence of nasopharyngitis was also significantly higher in 684 patients treated with anifrolumab (RR: 1.66, 95% CI: 1.25-2.20, P = 0.0004) and in the 300 mg subgroup (RR: 1.67, 95% CI: 1.19-2.35, P = 0.003). Additionally, bronchitis (RR: 1.96, 95% CI: 1.32-2.92, P = 0.0009) and cough (RR: 2.1, 95% CI: 1.0-4.40, P = 0.050) also occurred more frequently among patients receiving anifrolumab than among those receiving placebo. The incidence of bronchitis was higher in the 300 mg anifrolumab subgroup than in the placebo group (RR: 2.17, 95% CI: 1.33-3.54, P=0.002). Sinusitis was observed in 24 and 12 patients receiving anifrolumab or placebo, respectively, but there was no significant difference between the two groups (RR: 1.60, 95% CI: 0.85-3.01, P = 0.140).

### Infection-related adverse events

Among the 4 included RCTs, in addition to respiratory infections, infection-related adverse events also included herpes zoster, tuberculosis infection, urinary tract infection, influenza, nonopportunistic infection and opportunistic infection. Herpes zoster was one of the major adverse events reported with anifrolumab. The frequency of herpes zoster in patients receiving anifrolumab was significantly higher than that in patients receiving placebo (RR: 3.40, 95% CI: 1.90-6.07, P < 0.0001). Similar findings were found in the subgroup analysis. Herpes zoster occurred in 28 and 10 patients in the 300 mg subgroup (RR: 3.30, 95% CI: 1.57-6.95, P = 0.002) and 1000 mg subgroup (RR: 4.81, 95% CI: 1.08-21.41, P = 0.04) receiving anifrolumab, respectively. Other infection events included tuberculosis infection (RR: 2.09, 95% CI: 0.54-8.16, P = 0.29), opportunistic infection (RR: 0.84, 95% CI: 0.10-6.69, P=0.87), nonopportunistic infection (RR: 0.72, 95% CI: 0.39-1.35, P=0.31), urinary tract infection (RR: 0.83, 95% CI: 0.62-1.11, P=0.21) and influenza (RR: 1.62, 95% CI: 0.83-3.13, P=0.15), and there were no statistically significant differences between the anifrolumab and placebo groups.

### Other adverse events

In the MUSE (NCT01438489) phase II study, headache was one of the most frequently reported adverse events (13). In this study, headache occurred in 51 patients receiving anifrolumab and 29 receiving placebo, with no significant difference between the two groups (RR: 0.99, 95% CI: 0.69-1.42, P = 0.94). In addition, there was no significant difference in the incidence of adverse events of special interest, such as malignancy, anaphylaxis and infusion-related reactions, between patients treated with anifrolumab and placebo. Malignancy occurred in 4 patients receiving anifrolumab compared with 2 in the placebo group (RR: 1.54, 95% CI: 0.38-6.20, P=0.54). Anaphylaxis occurred in only one patient who received 150 mg of anifrolumab and none in the other patients (including placebo) (RR: 5.90, 95% CI: 0.24-143.54, P=0.28). A total of 657 patients who received intravenous anifrolumab experienced infusion reactions compared with 33 of 467 patients who received placebo (RR: 1.24, 95% CI: 0.87-1.79, P=0.24).

## Discussion

Anifrolumab is a fully humanized IFNAR inhibitor. Type 1 interferon can regulate the survival, activation and function of immune cells such as dendritic cells and T cells through a variety of different mechanisms, thereby promoting the occurrence and development of SLE (13–16). This study includes four currently available RCTs comparing anifrolumab to placebo in patients with moderate-to-severe systemic lupus erythematosus. The aim of this study was to strengthen the evidence in favor of anifrolumab and conduct a meta-analysis to investigate the relative risk of adverse events included in clinical trials. Our findings show that anifrolumab is safe in the treatment of SLE. Patients receiving anifrolumab had a lower incidence of serious adverse events than controls but an increased risk of infection, but with herpes zoster and respiratory tract infections being the most common, as discussed in more detail later. Of note, patients with active and severe lupus nephritis or neuropsychiatric SLE were excluded from these 4 studies.

The most frequent adverse events in SLE patients receiving anifrolumab included upper respiratory tract infection, nasopharyngitis, bronchitis, herpes zoster, headache, urinary tract infection, and infusion-related reactions, but most of these adverse events were mild or moderate intensity (7–10, 17, 18). The increased risk of infection was the main adverse event of anifrolumab, especially respiratory infection and herpes zoster. In this respect of increasing the risk of infection, anifrolumab has a similar incidence as that reported in clinical trials of other drugs used to treat SLE, such as monoclonal antibodies to B-cell activating factor or CD22 (17, 19, 20). In our meta-analysis, the incidence of adverse events such as herpes zoster, upper respiratory tract infection, nasopharyngitis, bronchitis and cough in patients treated with anifrolumab was higher than that in the control group. A recent meta-analysis also showed that the incidence of herpes zoster was significantly higher than that in the placebo group, which may be related to the mechanism of anifrolumab (11). Almost all herpes zoster events were cutaneous and rarely disseminated. In cases of herpes zoster, antiviral therapy was often effective and responsive. Previous study suggested that we can try to reduce the risk of herpes zoster recurrence by vaccination, but at the same time, vaccination also has the risk of inducing disease activity (21). Therefore, the best measure is to vaccinate the inactive subunit or inactivated herpes zoster vaccine. However, there is still a lack of in-depth research in this area. In addition, compared with the previous 52-week observational results, the frequency of herpes zoster did not increase in a 3-year open label extension trial (7–10, 22). This finding suggests that there is no correlation between the incidence of herpes zoster and the duration of anifrolumab treatment for SLE, and few patients stop anifrolumab treatment because of herpes zoster.

Moreover, this study found that there was no significant increase in the incidence of serious adverse events after receiving anifrolumab. Serious adverse events leading to discontinuation were rare. In all four RCTs, only three people died during treatment with anifrolumab, of which two died of pneumonia and one died of acute colitis. Compared with placebo, patients treated with anifrolumab had a higher incidence of anaphylaxis and infusion-related reactions, but most of them were mild or moderate. In addition, the incidence of tuberculosis infection and malignancy did not increase significantly. Additionally, previous studies have shown that with increasing doses of anifrolumab, the risk of infection also increases, but the effect of the treatment does not increase (7, 8, 11, 23). The results of the study further showed that compared with 150 mg and 1000 mg, the dose-benefit ratio of 300 mg was better. The latest pharmacokinetic study of anifrolumab and systemic lupus erythematosus also supports this conclusion (24). Ian N Bruce et al. (NCT02962960) found that subcutaneous injection of anifrolumab may be an alternative medication for patients through subcutaneous anifrolumab in 27 patients with SLE, which is consistent with previous intravenous administration studies in terms of efficacy and safety (10).

Among other adverse events, compared with placebo, there was no significant increase in the incidence of serious adverse events with anifrolumab. Serious adverse events leading to discontinuation of the trial drug were rare. In all four RCTs, only three people died during treatment with anifrolumab. Anaphylaxis and infusion-related reactions occurred at a higher frequency in patients receiving anifrolumab versus placebo but were mostly mild. In addition, the incidence of tuberculosis infection and malignancy did not increase significantly.

There are several weaknesses in this study. First, the limited number of included studies introduced the possibility of sampling error and publication bias. Second, the course of RCTs included in the study was only approximately 1 year, and there is a lack of longer-term observational reports. At the same time, RCTs and their long-term extension studies tend to sub-select patients with sustained response and do not include those who withdraw treatment due to intolerance. These study often like to exclude patients with severe comorbidities. In our inclusion of these studies, patients with severe lupus nephritis, lupus neuropsychiatric disorders and pediatrics were excluded. A recent phase II double-blind randomized controlled trial showed that anifrolumab can effectively reduce CRP levels in patients with grade III/IV lupus nephritis, but the primary endpoint of the trial (24-hour urine protein to creatinine ratio) was not met (25). Therefore, the efficacy and safety of anifrolumab in these subgroups remains to be studied and needs to be supplemented with real-world data.

## Conclusion

In conclusion, anifrolumab may be generally safe in the treatment of systemic lupus erythematosus. The best available dose for the clinical benefit of anifrolumab is 300 mg, whether subcutaneous or intravenous. And the incidence of drug-related serious adverse events is very low at this dose. The most common adverse events in patients receiving anifrolumab were herpes zoster, upper respiratory tract infection, nasopharyngitis, bronchitis, and cough. Anifrolumab is a potential option for the treatment of SLE, but the long-term efficacy and safety of the drug need more clinical studies.

## Data availability statement

The original contributions presented in the study are included in the article/**Supplementary Material**. Further inquiries can be directed to the corresponding author.

## Author contributions

ZL and RC conceptualized and designed the research, participated in data collection, extraction and interpretation, prepared forms, wrote and drafted the first draft, and approved the final draft, verified the analysis method, completed all relevant calculations and technical details of the meta-analysis, reviewed and modified the first draft, and approved the final draft submitted. YL participated in the interpretation of data, reviewed and modified the first draft, and approved the final manuscript submitted. All authors contributed to the article and approved the submitted version.

## Funding

This work was supported by the 1.3.5 Project for Disciplines of Excellence, West China Hospital, Sichuan University (No. ZYGD18015 and No.ZYJC18003).

## Conflict of interest

The authors declare that the research was conducted in the absence of any commercial or financial relationships that could be construed as a potential conflict of interest.

## Publisher’s note

All claims expressed in this article are solely those of the authors and do not necessarily represent those of their affiliated organizations, or those of the publisher, the editors and the reviewers. Any product that may be evaluated in this article, or claim that may be made by its manufacturer, is not guaranteed or endorsed by the publisher.

## Supplementary material

Supplementary Figure 1 Forest plot comparing adverse events of anifrolumab groups and the controls. “Experimental” refers to patients with SLE who received anifrolumab (150mg/300mg/1000mg), and “Control” refers to patients with SLE who received placebo. SLE, systemic lupus erythematosus;95% CI: 95% confidence interval.

## References

[1] Barber MRW Drenkard C Falasinnu T Hoi A Mak A Kow NY . Global epidemiology of systemic lupus erythematosus. Nat Rev Rheumatol (2021) 17(9):515–32. doi: 10.1038/s41584-021-00668-1 PMC8982275 34345022

[2] Tsokos GC . Systemic lupus erythematosus. N Engl J Med (2011) 365(22):2110–21. doi: 10.1056/NEJMra1100359 22129255

[3] Tanaka Y . State-of-the-art treatment of systemic lupus erythematosus. Int J Rheum Dis (2020) 23(4):465–71. doi: 10.1111/1756-185X.13817 PMC7187183 32134201

[4] Crow MK . Type I interferon in the pathogenesis of lupus. J Immunol (2014) 192(12):5459–68. doi: 10.4049/jimmunol.1002795 PMC4083591 24907379

[5] Niewold TB . Connective tissue diseases: Targeting type I interferon in systemic lupus erythematosus. Nat Rev Rheumatol (2016) 12(7):377–8. doi: 10.1038/nrrheum.2016.83 27225301

[6] Becker AM Dao KH Han BK Kornu R Lakhanpal S Mobley AB . SLE peripheral blood b cell, T cell and myeloid cell transcriptomes display unique profiles and each subset contributes to the interferon signature. PloS One (2013) 8(6):e67003. doi: 10.1371/journal.pone.0067003 23826184 PMC3691135

[7] Furie R Khamashta M Merrill JT Werth VP Kalunian K Brohawn P . Anifrolumab, an anti–interferon-α receptor monoclonal antibody, in moderate-to-Severe systemic lupus erythematosus. Arthritis Rheumatol (2017) 69(2):376–86. doi: 10.1002/art.39962 PMC5299497 28130918

[8] Furie RA Morand EF Bruce IN Manzi S Kalunian KC Vital EM . Type I interferon inhibitor anifrolumab in active systemic lupus erythematosus (TULIP-1): a randomised, controlled, phase 3 trial. Lancet Rheumatol (2019) 1(4):e208–19. doi: 10.1016/S2665-9913(19)30076-1 38229377

[9] Morand EF Furie R Tanaka Y Bruce IN Askanase AD Richez C . Trial of anifrolumab in active systemic lupus erythematosus. New Engl J Med (2020) 382(3):211–21. doi: 10.1056/NEJMoa1912196 31851795

[10] Bruce IN Nami A Schwetje E Pierson ME Rouse T Chia YL . Pharmacokinetics, pharmacodynamics, and safety of subcutaneous anifrolumab in patients with systemic lupus erythematosus, active skin disease, and high type I interferon gene signature: a multicentre, randomised, double-blind, placebo-controlled, phase 2 study. Lancet Rheumatol (2021) 3(2):e101–10. doi: 10.1016/S2665-9913(20)30342-8 38279367

[11] Lee YH Song GG . Anifrolumab for the treatment of active systemic lupus erythematosus: a meta-analysis of randomized controlled trials. Z fur Rheumatol (2020) 80(10):988–94. doi: 10.1007/s00393-020-00928-7 33216191

[12] Sterne JAC Savović J Page MJ Elbers RG Blencowe NS Boutron I . RoB 2: a revised tool for assessing risk of bias in randomised trials. BMJ (2019) 366:l4898. doi: 10.1136/bmj.l4898 31462531

[13] Bezalel S Guri KM Elbirt D Asher I Sthoeger ZM . Type I interferon signature in systemic lupus erythematosus. Isr Med Assoc J (2014) 16(4):246–9. 24834763

[14] Yan B Ye S Chen G Kuang M Shen N Chen S . Dysfunctional CD4+,CD25+ regulatory T cells in untreated active systemic lupus erythematosus secondary to interferon-alpha-producing antigen-presenting cells. Arthritis Rheumatol (2008) 58(3):801–12. doi: 10.1002/art.23268 18311820

[15] Andrade D Kim M Blanco LP Karumanchi SA Koo GC Redecha P . Interferon-α and angiogenic dysregulation in pregnant lupus patients who develop preeclampsia. Arthritis Rheumatol (2015) 67(4):977–87. doi: 10.1002/art.39029 PMC4380868 25603823

[16] Bekeredjian-Ding IB Wagner M Hornung V Giese T Schnurr M Endres S . Plasmacytoid dendritic cells control TLR7 sensitivity of naive b cells *via* type I IFN. J Immunol (2005) 174(7):4043–50. doi: 10.4049/jimmunol.174.7.4043 15778362

[17] Tummala R Abreu G Pineda L Michaels MA Kalyani RN Furie RA . Safety profile of anifrolumab in patients with active SLE: An integrated analysis of phase II and III trials. Lupus Sci Med (2021) 8(1):e000464. doi: 10.1136/lupus-2020-000464 33597205 PMC7893670

[18] Tanaka Y Takeuchi T Okada M Ishii T Nakajima H Kawai S . Safety and tolerability of anifrolumab, a monoclonal antibody targeting type I interferon receptor, in Japanese patients with systemic lupus erythematosus: A multicenter, phase 2, open-label study. Modern Rheumatol (2020) 30(1):101–8. doi: 10.1080/14397595.2019.1583833 30793642

[19] Clowse ME Wallace DJ Furie RA Petri MA Pike MC Leszczyń ski P . Efficacy and safety of epratuzumab in moderately to severely active systemic lupus erythematosus: Results from two phase III randomized, double-blind, placebo-controlled trials. Arthritis Rheumatol (Hoboken NJ) (2017) 69(2):362–75. doi: 10.1002/art.39856 PMC5299488 27598855

[20] Merrill JT van Vollenhoven RF Buyon JP Furie RA Stohl W Morgan-Cox M . Efficacy and safety of subcutaneous tabalumab, a monoclonal antibody to b-cell activating factor, in patients with systemic lupus erythematosus: results from ILLUMINATE-2, a 52-week, phase III, multicentre, randomised, double-blind, placebo-controlled study. Ann Rheum Dis (2016) 75(2):332–40. doi: 10.1136/annrheumdis-2015-207654 26293163

[21] Mok CC . Herpes zoster vaccination in systemic lupus erythematosus: the current status. Hum Vaccin Immunother (2019) 15(1):45–8. doi: 10.1080/21645515.2018.1514228 PMC6363132 30130445

[22] Chatham WW Furie R Saxena A Brohawn P Schwetje E Abreu G . Long-term safety and efficacy of anifrolumab in adults with systemic lupus erythematosus: Results of a phase II open-label extension study. Arthritis Rheumatol (2021) 73(5):816–25. doi: 10.1002/art.41598 PMC8252065 33225631

[23] Chia YL Santiago L Wang B Kuruvilla D Wang S Tummala R . Exposure-response analysis for selection of optimal dosage regimen of anifrolumab in patients with systemic lupus erythematosus. Rheumatology (Oxford) (2021) 60(12):5854–62. doi: 10.1093/rheumatology/keab176 33629110

[24] Chia YL Zhang J Tummala R Rouse T Furie RA Morand EF . Relationship of anifrolumab pharmacokinetics with efficacy and safety in patients with systemic lupus erythematosus. Rheumatol (Oxford) (2022) 61(5):1900–10. doi: 10.1093/rheumatology/keab704 PMC9071514 34528084

[25] Jayne D Rovin B Mysler EF Furie RA Houssiau FA Trasieva T . Phase II randomised trial of type I interferon inhibitor anifrolumab in patients with active lupus nephritis. Ann Rheum Dis (2022) 81(4):496–506. doi: 10.1136/annrheumdis-2021-221478 35144924 PMC8921596

