# Supplementary material for: Evaluation of anifrolumab safety in systemic lupus erythematosus: A meta-analysis and systematic review
Source: Front Immunol. 2022 Sep 23;13:996662. doi: 10.3389/fimmu.2022.996662 (PMC9537685; doi:10.3389/fimmu.2022.996662)

1.1 serious adverse events

Study or Subgroup	Experimental Events	Experimental Total	Control Events	Control Total	Weight	Risk Ratio M-H, Fixed, 95% CI	Year
**1.1.1 150mg**							
Richard A. Furie 2019	10	93	30	184	16.8%	0.66 [0.34, 1.29]	2019
Ian N Bruce 2021	4	14	0	9	0.5%	6.00 [0.36, 99.66]	2021
**Subtotal (95% CI)**		**107**		**193**	**17.3%**	**0.81 [0.44, 1.52]**	
Total events	14		30				
Heterogeneity: Chi² = 2.32, df = 1 (P = 0.13); I² = 57%							
Test for overall effect: Z = 0.64 (P = 0.52)							
**1.1.2 300mg**							
Richard Furie 2017	16	99	19	101	15.7%	0.86 [0.47, 1.57]	2017
Richard A. Furie 2019	25	180	30	184	24.7%	0.85 [0.52, 1.39]	2019
E.F. Morand 2020	15	180	31	182	25.7%	0.49 [0.27, 0.87]	2020
Ian N Bruce 2021	2	13	0	9	0.5%	3.57 [0.19, 66.61]	2021
**Subtotal (95% CI)**		**472**		**476**	**66.6%**	**0.73 [0.54, 1.00]**	
Total events	58		80				
Heterogeneity: Chi² = 3.61, df = 3 (P = 0.31); I² = 17%							
Test for overall effect: Z = 1.94 (P = 0.05)							
**1.1.3 1000mg**							
Richard Furie 2017	18	105	19	101	16.1%	0.91 [0.51, 1.63]	2017
**Subtotal (95% CI)**		**105**		**101**	**16.1%**	**0.91 [0.51, 1.63]**	
Total events	18		19				
Heterogeneity: Not applicable							
Test for overall effect: Z = 0.31 (P = 0.76)							
**Total (95% CI)**		**684**		**770**	**100.0%**	**0.78 [0.60, 1.00]**	
Total events	90		129				
Heterogeneity: Chi² = 6.27, df = 6 (P = 0.39); I² = 4%							
Test for overall effect: Z = 1.97 (P = 0.05)							
Test for subgroup differences: Chi² = 0.44, df = 2 (P = 0.80), I² = 0%							

Risk Ratio M-H, Fixed, 95% CI

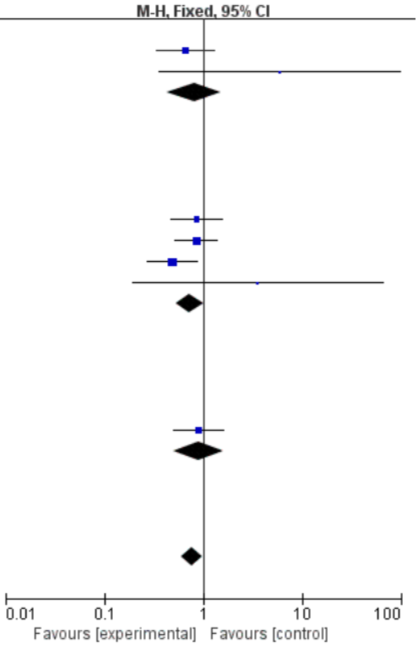

1.2 death

Study or Subgroup	Experimental Events	Experimental Total	Control Events	Control Total	Weight	Risk Ratio M-H, Fixed, 95% CI	Year
**1.2.1 150mg**							
Richard A. Furie 2019	0	93	0	184		Not estimable	2019
Ian N Bruce 2021	0	14	0	9		Not estimable	2021
**Subtotal (95% CI)**		**107**		**193**		**Not estimable**	
Total events	0		0				
Heterogeneity: Not applicable							
Test for overall effect: Not applicable							
**1.2.2 300mg**							
Richard Furie 2017	0	99	0	101		Not estimable	2017
Richard A. Furie 2019	1	180	0	184	32.9%	3.07 [0.13, 74.78]	2019
E.F. Morand 2020	1	180	0	182	33.1%	3.03 [0.12, 73.97]	2020
Ian N Bruce 2021	0	13	0	9		Not estimable	2021
**Subtotal (95% CI)**		**472**		**476**	**66.1%**	**3.05 [0.32, 29.18]**	
Total events	2		0				
Heterogeneity: Chi² = 0.00, df = 1 (P = 1.00); I² = 0%							
Test for overall effect: Z = 0.97 (P = 0.33)							
**1.2.3 1000mg**							
Richard Furie 2017	1	105	0	101	33.9%	2.89 [0.12, 70.05]	2017
**Subtotal (95% CI)**		**105**		**101**	**33.9%**	**2.89 [0.12, 70.05]**	
Total events	1		0				
Heterogeneity: Not applicable							
Test for overall effect: Z = 0.65 (P = 0.51)							
**Total (95% CI)**		**684**		**770**	**100.0%**	**2.99 [0.47, 18.91]**	
Total events	3		0				
Heterogeneity: Chi² = 0.00, df = 2 (P = 1.00); I² = 0%							
Test for overall effect: Z = 1.17 (P = 0.24)							
Test for subgroup differences: Chi² = 0.00, df = 1 (P = 0.98), I² = 0%							

Risk Ratio M-H, Fixed, 95% CI

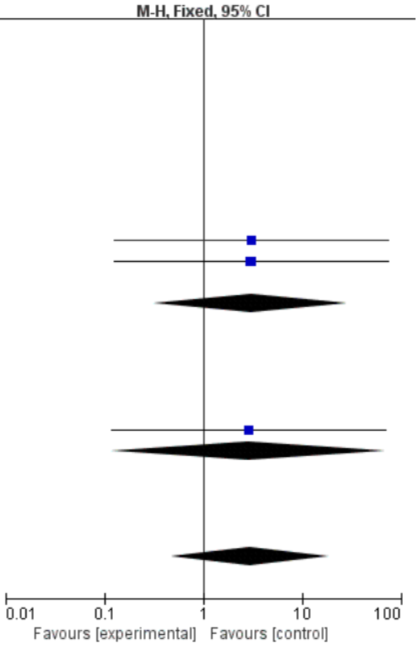

1.3 Adverse events leading to discontinuation

Study or Subgroup	Experimental Events	Experimental Total	Control Events	Control Total	Weight	Risk Ratio M-H, Fixed, 95% CI	Year
**1.3.1 150mg**							
Richard A. Furie 2019	5	93	5	184	8.7%	1.98 [0.59, 6.66]	2019
Ian N Bruce 2021	2	14	0	9	1.6%	3.33 [0.18, 62.37]	2021
**Subtotal (95% CI)**		**107**		**193**	**10.3%**	**2.18 [0.71, 6.72]**	
Total events	7		5				
Heterogeneity: Chi² = 0.11, df = 1 (P = 0.75); I² = 0%							
Test for overall effect: Z = 1.36 (P = 0.17)							
**1.3.2 300mg**							
Richard Furie 2017	3	99	8	101	20.6%	0.38 [0.10, 1.40]	2017
Richard A. Furie 2019	11	180	5	184	12.8%	2.25 [0.80, 6.34]	2019
E.F. Morand 2020	5	180	13	182	33.6%	0.39 [0.14, 1.07]	2020
Ian N Bruce 2021	3	13	0	9	1.5%	5.00 [0.29, 86.43]	2021
**Subtotal (95% CI)**		**472**		**476**	**68.5%**	**0.84 [0.48, 1.46]**	
Total events	22		26				
Heterogeneity: Chi² = 8.61, df = 3 (P = 0.03); I² = 65%							
Test for overall effect: Z = 0.63 (P = 0.53)							
**1.3.3 1000mg**							
Richard Furie 2017	10	105	8	101	21.2%	1.20 [0.49, 2.92]	2017
**Subtotal (95% CI)**		**105**		**101**	**21.2%**	**1.20 [0.49, 2.92]**	
Total events	10		8				
Heterogeneity: Not applicable							
Test for overall effect: Z = 0.41 (P = 0.68)							
**Total (95% CI)**		**684**		**770**	**100.0%**	**1.05 [0.69, 1.62]**	
Total events	39		39				
Heterogeneity: Chi² = 10.99, df = 6 (P = 0.09); I² = 45%							
Test for overall effect: Z = 0.24 (P = 0.81)							
Test for subgroup differences: Chi² = 2.36, df = 2 (P = 0.31), I² = 15.1%							

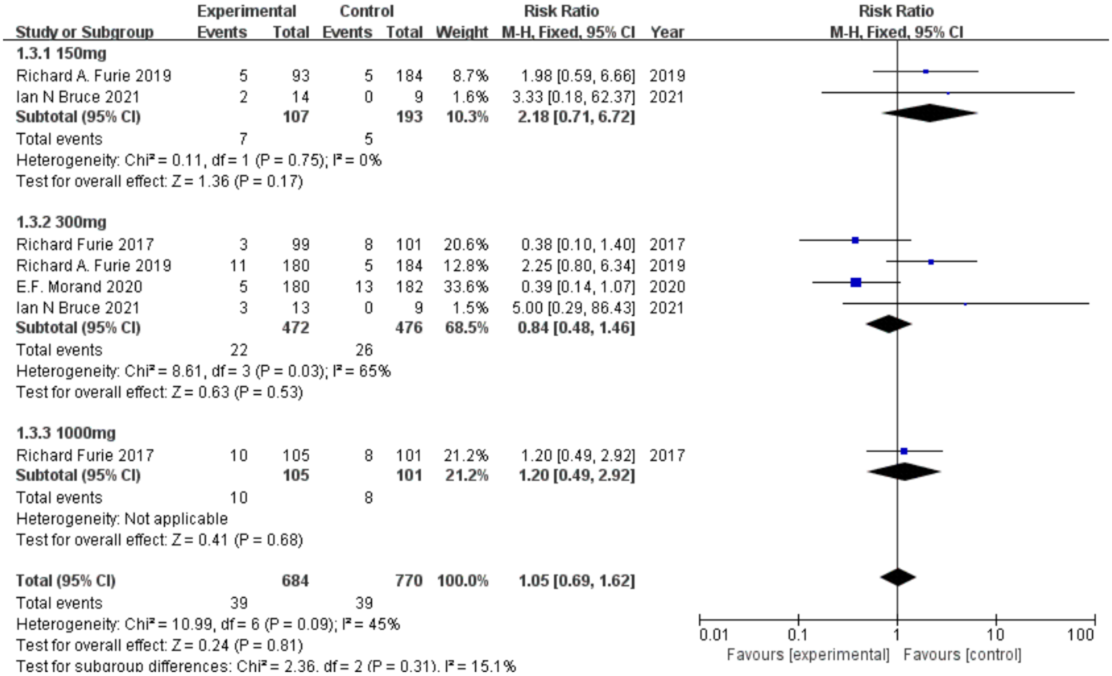

1.4 headache

Study or Subgroup	Experimental Events	Experimental Total	Control Events	Control Total	Weight	Risk Ratio M-H, Fixed, 95% CI	Year
**1.4.1 150mg**							
Richard A. Furie 2019	6	93	16	184	19.9%	0.74 [0.30, 1.83]	2019
Ian N Bruce 2021	2	14	0	9	1.1%	3.33 [0.18, 62.37]	2021
**Subtotal (95% CI)**		**107**		**193**	**21.1%**	**0.88 [0.38, 2.04]**	
Total events	8		16				
Heterogeneity: Chi² = 0.93, df = 1 (P = 0.33); I² = 0%							
Test for overall effect: Z = 0.30 (P = 0.76)							
**1.4.2 300mg**							
Richard Furie 2017	12	99	13	101	23.9%	0.94 [0.45, 1.96]	2017
Richard A. Furie 2019	17	180	16	184	29.4%	1.09 [0.57, 2.08]	2019
Ian N Bruce 2021	2	13	0	9	1.1%	3.57 [0.19, 66.61]	2021
**Subtotal (95% CI)**		**292**		**294**	**54.3%**	**1.07 [0.66, 1.73]**	
Total events	31		29				
Heterogeneity: Chi² = 0.77, df = 2 (P = 0.68); I² = 0%							
Test for overall effect: Z = 0.29 (P = 0.77)							
**1.4.3 1000mg**							
Richard Furie 2017	12	105	13	101	24.6%	0.89 [0.43, 1.85]	2017
**Subtotal (95% CI)**		**105**		**101**	**24.6%**	**0.89 [0.43, 1.85]**	
Total events	12		13				
Heterogeneity: Not applicable							
Test for overall effect: Z = 0.32 (P = 0.75)							
**Total (95% CI)**		**504**		**588**	**100.0%**	**0.99 [0.69, 1.42]**	
Total events	51		58				
Heterogeneity: Chi² = 1.97, df = 5 (P = 0.85); I² = 0%							
Test for overall effect: Z = 0.08 (P = 0.94)							
Test for subgroup differences: Chi² = 0.27, df = 2 (P = 0.87), I² = 0%							

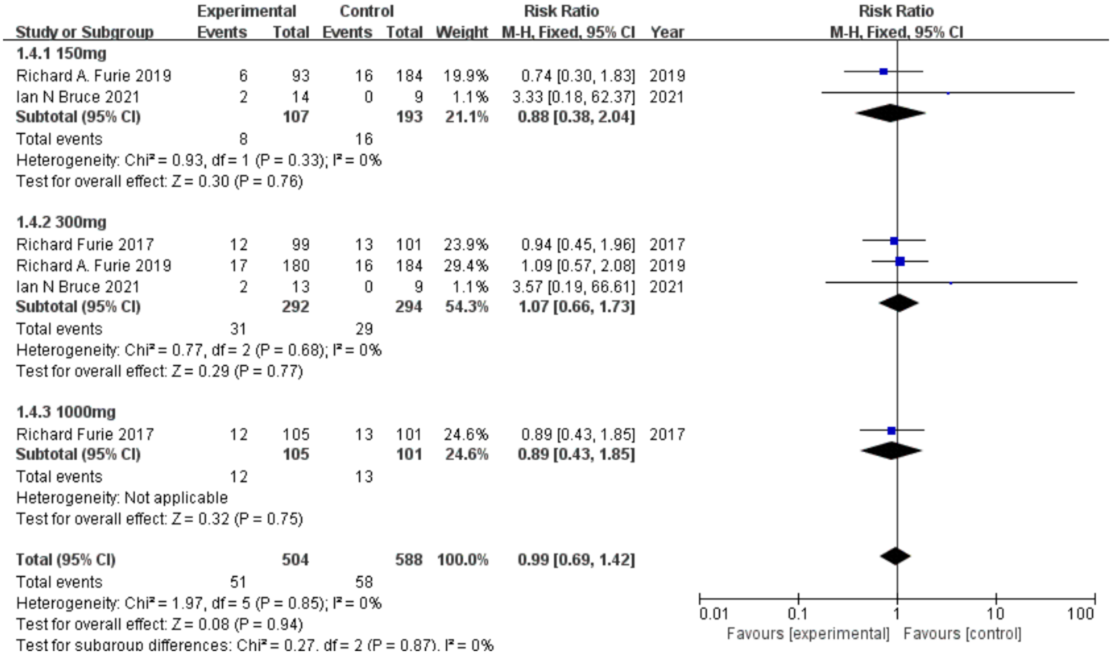

1.5 Upper respiratory tract infection

Study or Subgroup	Experimental Events	Experimental Total	Control Events	Control Total	Weight	Risk Ratio M-H, Fixed, 95% CI	Year
**1.5.1 150mg**							
Richard A. Furie 2019	16	93	18	184	16.1%	1.76 [0.94, 3.29]	2019
Ian N Bruce 2021	5	14	3	9	4.9%	1.07 [0.34, 3.42]	2021
**Subtotal (95% CI)**		**107**		**193**	**21.0%**	**1.60 [0.92, 2.77]**	
Total events	21		21				
Heterogeneity: Chi² = 0.55, df = 1 (P = 0.46); I² = 0%							
Test for overall effect: Z = 1.67 (P = 0.09)							
**1.5.2 300mg**							
Richard Furie 2017	13	99	10	101	13.2%	1.33 [0.61, 2.88]	2017
Richard A. Furie 2019	22	180	18	184	23.7%	1.25 [0.69, 2.25]	2019
E.F. Morand 2020	39	180	18	182	23.8%	2.19 [1.30, 3.68]	2020
Ian N Bruce 2021	1	13	3	9	4.7%	0.23 [0.03, 1.88]	2021
**Subtotal (95% CI)**		**472**		**476**	**65.5%**	**1.53 [1.10, 2.15]**	
Total events	75		49				
Heterogeneity: Chi² = 5.55, df = 3 (P = 0.14); I² = 46%							
Test for overall effect: Z = 2.50 (P = 0.01)							
**1.5.3 1000mg**							
Richard Furie 2017	11	105	10	101	13.6%	1.06 [0.47, 2.38]	2017
**Subtotal (95% CI)**		**105**		**101**	**13.6%**	**1.06 [0.47, 2.38]**	
Total events	11		10				
Heterogeneity: Not applicable							
Test for overall effect: Z = 0.14 (P = 0.89)							
**Total (95% CI)**		**684**		**770**	**100.0%**	**1.48 [1.13, 1.94]**	
Total events	107		80				
Heterogeneity: Chi² = 6.85, df = 6 (P = 0.34); I² = 12%							
Test for overall effect: Z = 2.86 (P = 0.004)							
Test for subgroup differences: Chi² = 0.78, df = 2 (P = 0.68), I² = 0%							

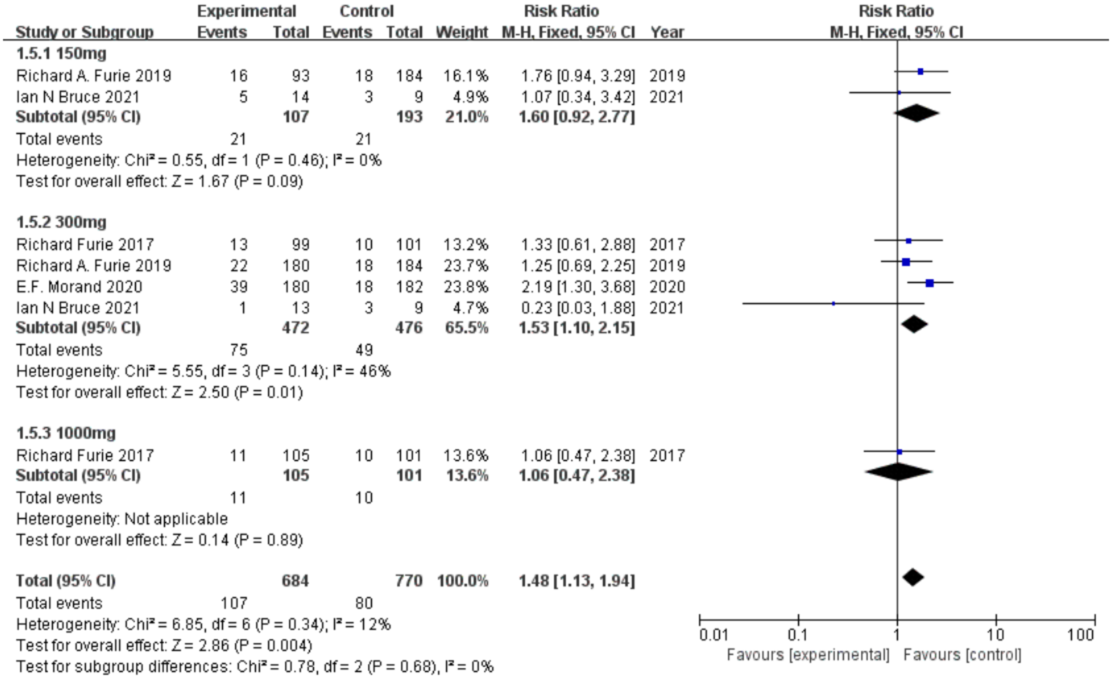

1.6 Nasopharyngitis

Study or Subgroup	Experimental Events	Experimental Total	Control Events	Control Total	Weight	Risk Ratio M-H, Fixed, 95% CI	Year
**1.6.1 150mg**							
Richard A. Furie 2019	14	93	22	184	22.1%	1.26 [0.68, 2.35]	2019
Ian N Bruce 2021	3	14	1	9	1.8%	1.93 [0.24, 15.79]	2021
**Subtotal (95% CI)**		**107**		**193**	**23.9%**	**1.31 [0.72, 2.38]**	
Total events	17		23				
Heterogeneity: Chi² = 0.15, df = 1 (P = 0.70); I² = 0%							
Test for overall effect: Z = 0.89 (P = 0.37)							
**1.6.2 300mg**							
Richard Furie 2017	12	99	4	101	5.9%	3.06 [1.02, 9.17]	2017
Richard A. Furie 2019	36	180	22	184	32.5%	1.67 [1.03, 2.73]	2019
E.F. Morand 2020	28	180	20	182	29.7%	1.42 [0.83, 2.42]	2020
Ian N Bruce 2021	2	13	1	9	1.8%	1.38 [0.15, 13.07]	2021
**Subtotal (95% CI)**		**472**		**476**	**70.0%**	**1.67 [1.19, 2.35]**	
Total events	78		47				
Heterogeneity: Chi² = 1.57, df = 3 (P = 0.67); I² = 0%							
Test for overall effect: Z = 2.99 (P = 0.003)							
**1.6.3 1000mg**							
Richard Furie 2017	12	105	4	101	6.1%	2.89 [0.96, 8.65]	2017
**Subtotal (95% CI)**		**105**		**101**	**6.1%**	**2.89 [0.96, 8.65]**	
Total events	12		4				
Heterogeneity: Not applicable							
Test for overall effect: Z = 1.89 (P = 0.06)							
**Total (95% CI)**		**684**		**770**	**100.0%**	**1.66 [1.25, 2.20]**	
Total events	107		74				
Heterogeneity: Chi² = 3.31, df = 6 (P = 0.77); I² = 0%							
Test for overall effect: Z = 3.51 (P = 0.0004)							
Test for subgroup differences: Chi² = 1.58, df = 2 (P = 0.45), I² = 0%							

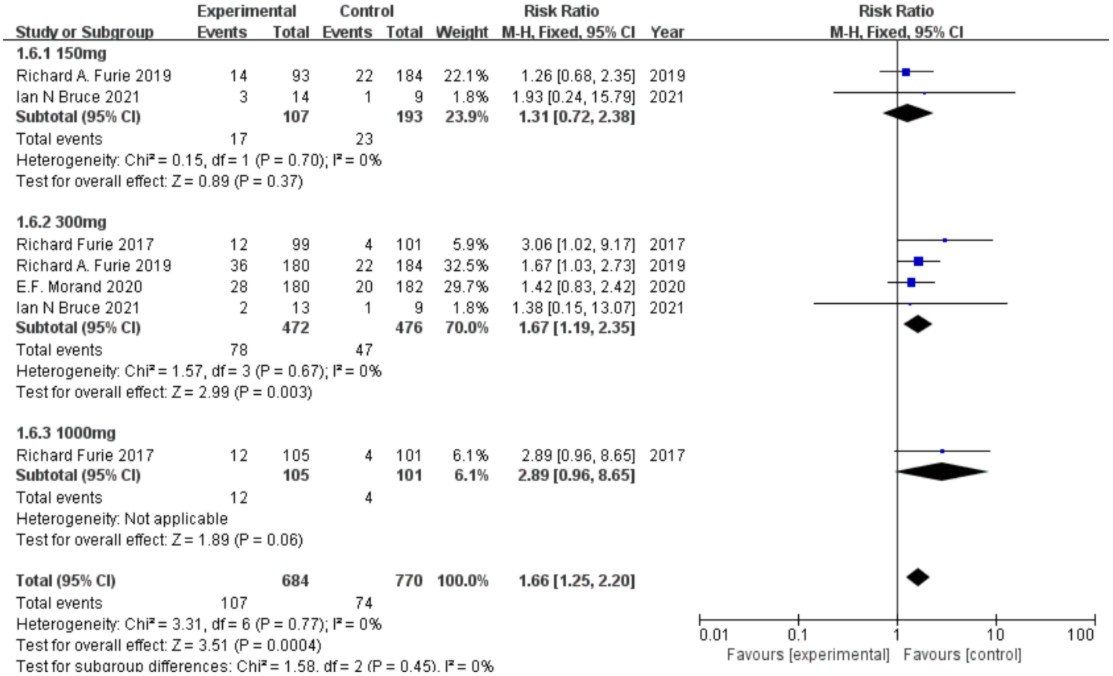

1.7 Urinary tract infection

Study or Subgroup	Experimental Events	Experimental Total	Control Events	Control Total	Weight	Risk Ratio M-H, Fixed, 95% CI	Year
**1.7.1 150mg**							
Richard A. Furie 2019	9	93	27	184	19.7%	0.66 [0.32, 1.34]	2019
**Subtotal (95% CI)**		**93**		**184**	**19.7%**	**0.66 [0.32, 1.34]**	
Total events	9		27				
Heterogeneity: Not applicable							
Test for overall effect: Z = 1.15 (P = 0.25)							
**1.7.2 300mg**							
Richard Furie 2017	15	99	11	101	11.9%	1.39 [0.67, 2.88]	2017
Richard A. Furie 2019	22	180	27	184	29.1%	0.83 [0.49, 1.41]	2019
E.F. Morand 2020	20	180	25	182	27.1%	0.81 [0.47, 1.40]	2020
**Subtotal (95% CI)**		**459**		**467**	**68.0%**	**0.92 [0.66, 1.29]**	
Total events	57		63				
Heterogeneity: Chi² = 1.59, df = 2 (P = 0.45); I² = 0%							
Test for overall effect: Z = 0.48 (P = 0.63)							
**1.7.3 1000mg**							
Richard Furie 2017	7	105	11	101	12.2%	0.61 [0.25, 1.52]	2017
**Subtotal (95% CI)**		**105**		**101**	**12.2%**	**0.61 [0.25, 1.52]**	
Total events	7		11				
Heterogeneity: Not applicable							
Test for overall effect: Z = 1.06 (P = 0.29)							
**Total (95% CI)**		**657**		**752**	**100.0%**	**0.83 [0.62, 1.11]**	
Total events	73		101				
Heterogeneity: Chi² = 2.78, df = 4 (P = 0.60); I² = 0%							
Test for overall effect: Z = 1.26 (P = 0.21)							
Test for subgroup differences: Chi² = 1.20, df = 2 (P = 0.55), I² = 0%							

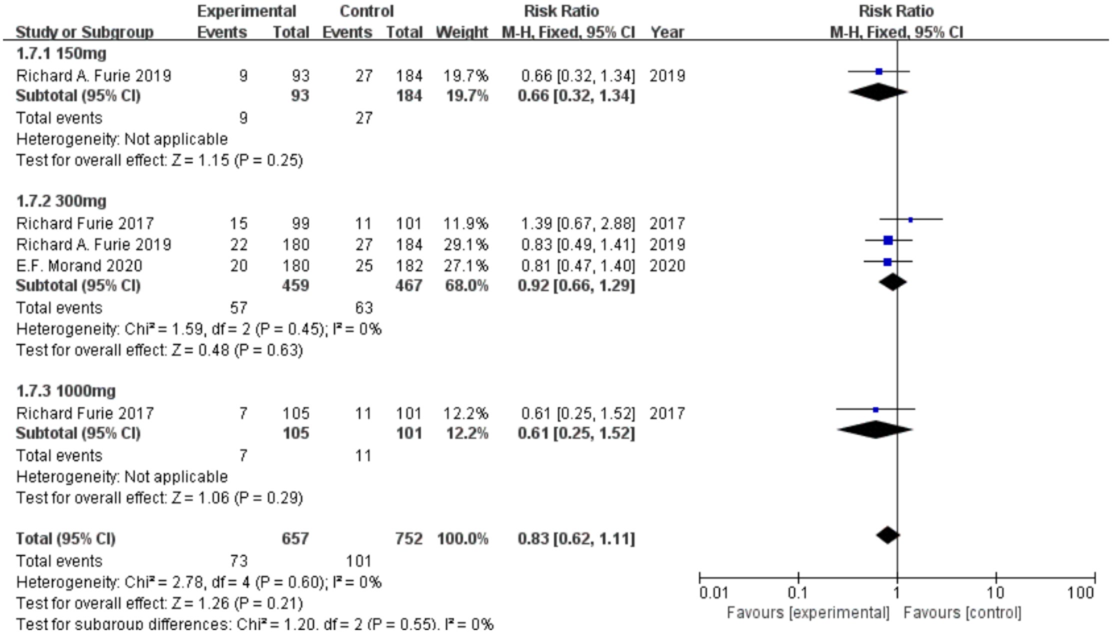

1.8 Bronchitis

Study or Subgroup	Experimental Events	Experimental Total	Control Events	Control Total	Weight	Risk Ratio M-H, Fixed, 95% CI	Year
**1.8.1 150mg**							
Richard A. Furie 2019	7	93	10	184	19.7%	1.38 [0.54, 3.52]	2019
Ian N Bruce 2021	1	14	1	9	3.6%	0.64 [0.05, 9.03]	2021
**Subtotal (95% CI)**		**107**		**193**	**23.3%**	**1.27 [0.53, 3.04]**	
Total events	8		11				
Heterogeneity: Chi² = 0.29, df = 1 (P = 0.59); I² = 0%							
Test for overall effect: Z = 0.54 (P = 0.59)							
**1.8.2 300mg**							
Richard Furie 2017	7	99	4	101	11.6%	1.79 [0.54, 5.91]	2017
Richard A. Furie 2019	16	180	10	184	29.1%	1.64 [0.76, 3.51]	2019
E.F. Morand 2020	22	180	7	182	20.5%	3.18 [1.39, 7.25]	2020
Ian N Bruce 2021	3	13	1	9	3.5%	2.08 [0.25, 16.92]	2021
**Subtotal (95% CI)**		**472**		**476**	**64.7%**	**2.17 [1.33, 3.54]**	
Total events	48		22				
Heterogeneity: Chi² = 1.45, df = 3 (P = 0.69); I² = 0%							
Test for overall effect: Z = 3.12 (P = 0.002)							
**1.8.3 1000mg**							
Richard Furie 2017	9	105	4	101	12.0%	2.16 [0.69, 6.81]	2017
**Subtotal (95% CI)**		**105**		**101**	**12.0%**	**2.16 [0.69, 6.81]**	
Total events	9		4				
Heterogeneity: Not applicable							
Test for overall effect: Z = 1.32 (P = 0.19)							
**Total (95% CI)**		**684**		**770**	**100.0%**	**1.96 [1.32, 2.92]**	
Total events	65		37				
Heterogeneity: Chi² = 2.81, df = 6 (P = 0.83); I² = 0%							
Test for overall effect: Z = 3.32 (P = 0.0009)							
Test for subgroup differences: Chi² = 1.14, df = 2 (P = 0.56), I² = 0%							

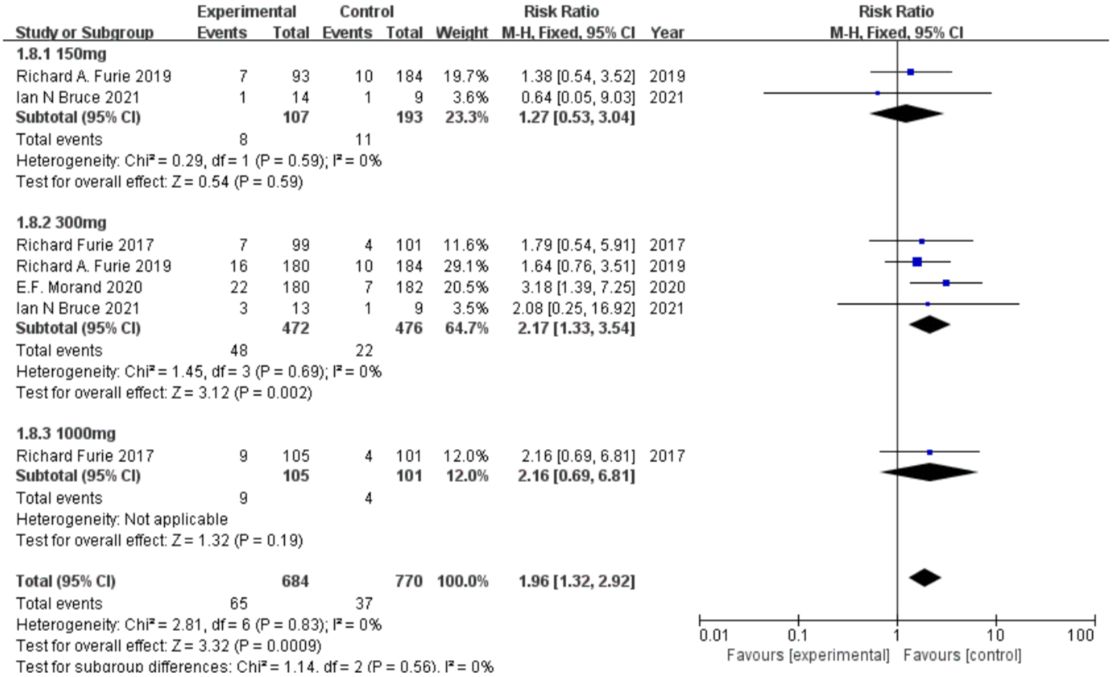

1.9 Herpes zoster

Study or Subgroup	Experimental Events	Experimental Total	Control Events	Control Total	Weight	Risk Ratio M-H, Fixed, 95% CI	Year
**1.9.1 150mg**							
Richard A. Furie 2019	5	93	3	184	14.4%	3.30 [0.81, 13.50]	2019
Ian N Bruce 2021	3	14	1	9	8.7%	1.93 [0.24, 15.79]	2021
**Subtotal (95% CI)**		**107**		**193**	**23.2%**	**2.78 [0.87, 8.89]**	
Total events	8		4				
Heterogeneity: Chi² = 0.17, df = 1 (P = 0.68); I² = 0%							
Test for overall effect: Z = 1.73 (P = 0.08)							
**1.9.2 300mg**							
Richard Furie 2017	5	99	2	101	14.2%	2.55 [0.51, 12.84]	2017
Richard A. Furie 2019	10	180	3	184	21.3%	3.41 [0.95, 12.18]	2019
E.F. Morand 2020	13	180	2	182	14.3%	6.57 [1.50, 28.71]	2020
Ian N Bruce 2021	0	13	1	9	12.5%	0.24 [0.01, 5.26]	2021
**Subtotal (95% CI)**		**472**		**476**	**62.2%**	**3.30 [1.57, 6.95]**	
Total events	28		8				
Heterogeneity: Chi² = 3.71, df = 3 (P = 0.29); I² = 19%							
Test for overall effect: Z = 3.14 (P = 0.002)							
**1.9.3 1000mg**							
Richard Furie 2017	10	105	2	101	14.6%	4.81 [1.08, 21.41]	2017
**Subtotal (95% CI)**		**105**		**101**	**14.6%**	**4.81 [1.08, 21.41]**	
Total events	10		2				
Heterogeneity: Not applicable							
Test for overall effect: Z = 2.06 (P = 0.04)							
**Total (95% CI)**		**684**		**770**	**100.0%**	**3.40 [1.90, 6.07]**	
Total events	46		14				
Heterogeneity: Chi² = 4.21, df = 6 (P = 0.65); I² = 0%							
Test for overall effect: Z = 4.14 (P < 0.0001)							
Test for subgroup differences: Chi² = 0.33, df = 2 (P = 0.85), I² = 0%							

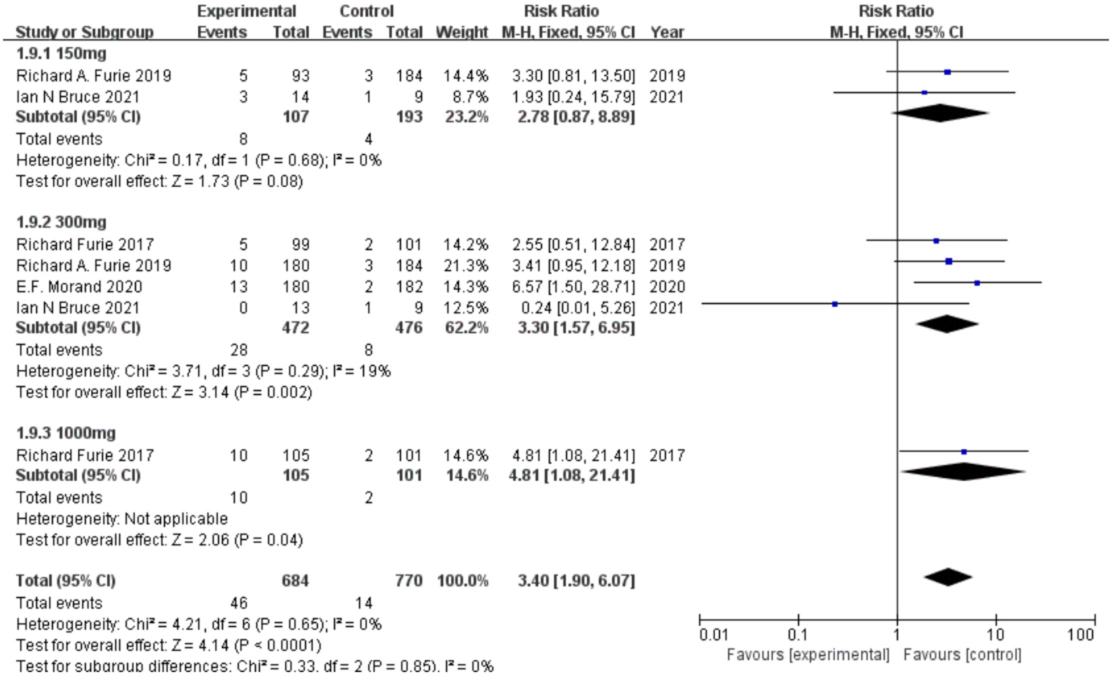

1.10 Influenza

Study or Subgroup	Experimental Events	Experimental Total	Control Events	Control Total	Weight	Risk Ratio M-H, Fixed, 95% CI	Year
**1.10.1 150mg**							
Richard A. Furie 2019	1	93	2	184	9.7%	0.99 [0.09, 10.77]	2019
Ian N Bruce 2021	1	14	0	9	4.3%	2.00 [0.09, 44.35]	2021
**Subtotal (95% CI)**		**107**		**193**	**14.0%**	**1.30 [0.20, 8.40]**	
Total events	2		2				
Heterogeneity: Chi² = 0.12, df = 1 (P = 0.72); I² = 0%							
Test for overall effect: Z = 0.28 (P = 0.78)							
**1.10.2 300mg**							
Richard Furie 2017	6	99	2	101	14.2%	3.06 [0.63, 14.80]	2017
Richard A. Furie 2019	2	180	2	184	14.2%	1.02 [0.15, 7.18]	2019
E.F. Morand 2020	4	180	6	182	42.9%	0.67 [0.19, 2.35]	2020
Ian N Bruce 2021	0	13	0	9		Not estimable	2021
**Subtotal (95% CI)**		**472**		**476**	**71.4%**	**1.22 [0.53, 2.79]**	
Total events	12		10				
Heterogeneity: Chi² = 2.21, df = 2 (P = 0.33); I² = 9%							
Test for overall effect: Z = 0.47 (P = 0.64)							
**1.10.3 1000mg**							
Richard Furie 2017	8	105	2	101	14.7%	3.85 [0.84, 17.68]	2017
**Subtotal (95% CI)**		**105**		**101**	**14.7%**	**3.85 [0.84, 17.68]**	
Total events	8		2				
Heterogeneity: Not applicable							
Test for overall effect: Z = 1.73 (P = 0.08)							
**Total (95% CI)**		**684**		**770**	**100.0%**	**1.62 [0.83, 3.13]**	
Total events	22		14				
Heterogeneity: Chi² = 4.15, df = 5 (P = 0.53); I² = 0%							
Test for overall effect: Z = 1.42 (P = 0.15)							
Test for subgroup differences: Chi² = 1.72, df = 2 (P = 0.42), I² = 0%							

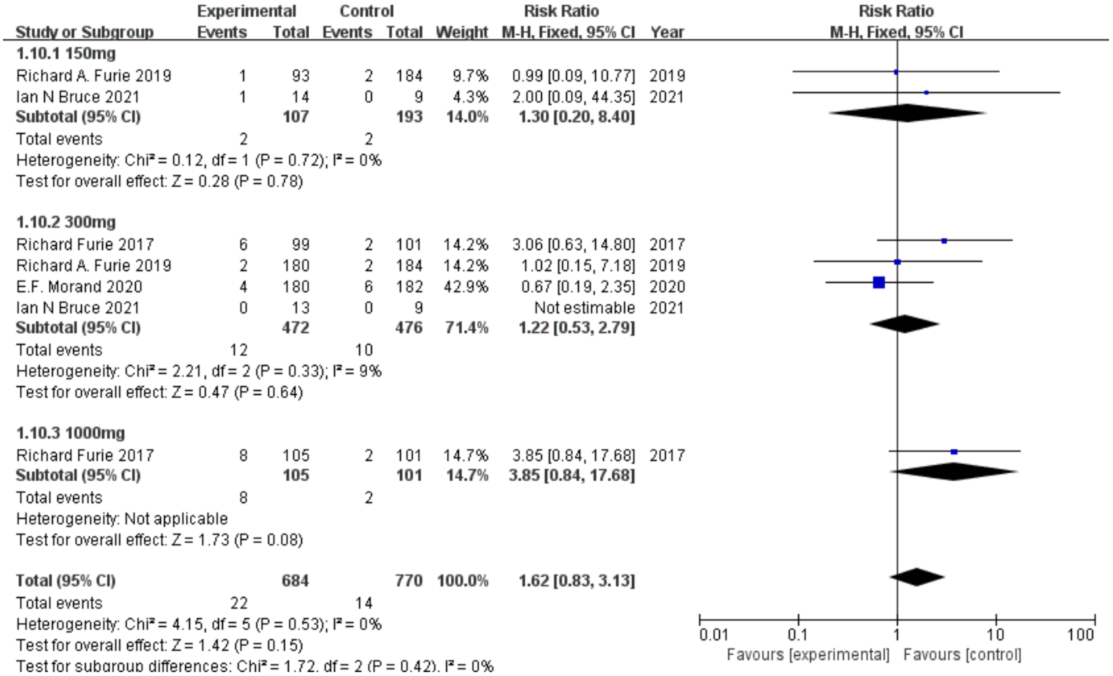

1.11 Sinusitis

Study or Subgroup	Experimental Events	Experimental Total	Control Events	Control Total	Weight	Risk Ratio M-H, Fixed, 95% CI	Year
**1.11.1 300mg**							
Richard Furie 2017	6	99	3	101	19.8%	2.04 [0.52, 7.93]	2017
E.F. Morand 2020	12	180	9	182	59.8%	1.35 [0.58, 3.12]	2020
**Subtotal (95% CI)**		**279**		**283**	**79.6%**	**1.52 [0.75, 3.10]**	
Total events	18		12				
Heterogeneity: Chi² = 0.26, df = 1 (P = 0.61); I² = 0%							
Test for overall effect: Z = 1.16 (P = 0.25)							
**1.11.2 1000mg**							
Richard Furie 2017	6	105	3	101	20.4%	1.92 [0.49, 7.49]	2017
**Subtotal (95% CI)**		**105**		**101**	**20.4%**	**1.92 [0.49, 7.49]**	
Total events	6		3				
Heterogeneity: Not applicable							
Test for overall effect: Z = 0.94 (P = 0.35)							
**Total (95% CI)**		**384**		**384**	**100.0%**	**1.60 [0.85, 3.01]**	
Total events	24		15				
Heterogeneity: Chi² = 0.35, df = 2 (P = 0.84); I² = 0%							
Test for overall effect: Z = 1.47 (P = 0.14)							
Test for subgroup differences: Chi² = 0.09, df = 1 (P = 0.76), I² = 0%							

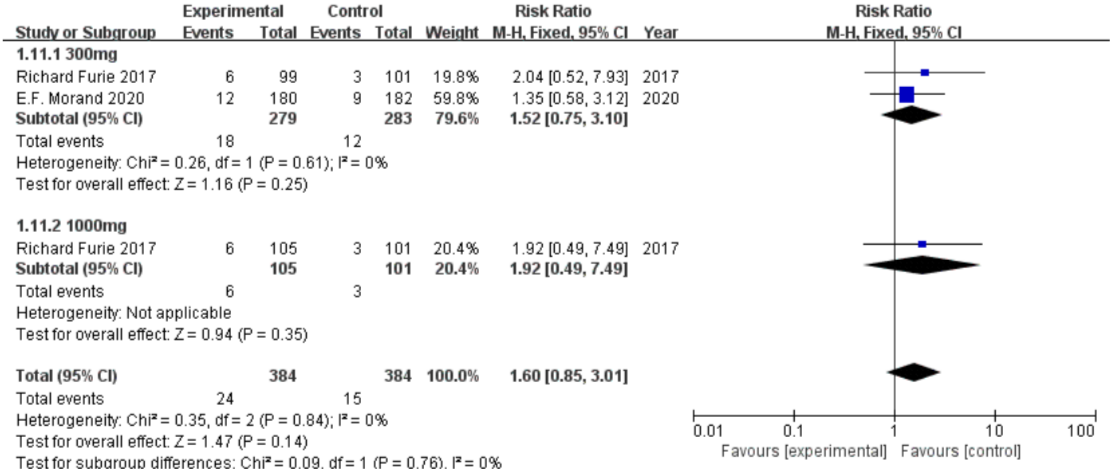

1.12 Cough

Study or Subgroup	Experimental Events	Experimental Total	Control Events	Control Total	Weight	Risk Ratio M-H, Fixed, 95% CI	Year
**1.12.1 300mg**							
Richard Furie 2017	3	99	2	101	19.8%	1.53 [0.26, 8.96]	2017
E.F. Morand 2020	10	180	6	182	59.8%	1.69 [0.63, 4.54]	2020
**Subtotal (95% CI)**		**279**		**283**	**79.6%**	**1.65 [0.69, 3.91]**	
Total events	13		8				
Heterogeneity: Chi² = 0.01, df = 1 (P = 0.93); I² = 0%							
Test for overall effect: Z = 1.13 (P = 0.26)							
**1.12.2 1000mg**							
Richard Furie 2017	8	105	2	101	20.4%	3.85 [0.84, 17.68]	2017
**Subtotal (95% CI)**		**105**		**101**	**20.4%**	**3.85 [0.84, 17.68]**	
Total events	8		2				
Heterogeneity: Not applicable							
Test for overall effect: Z = 1.73 (P = 0.08)							
**Total (95% CI)**		**384**		**384**	**100.0%**	**2.10 [1.00, 4.40]**	
Total events	21		10				
Heterogeneity: Chi² = 0.92, df = 2 (P = 0.63); I² = 0%							
Test for overall effect: Z = 1.96 (P = 0.05)							
Test for subgroup differences: Chi² = 0.90, df = 1 (P = 0.34), I² = 0%							

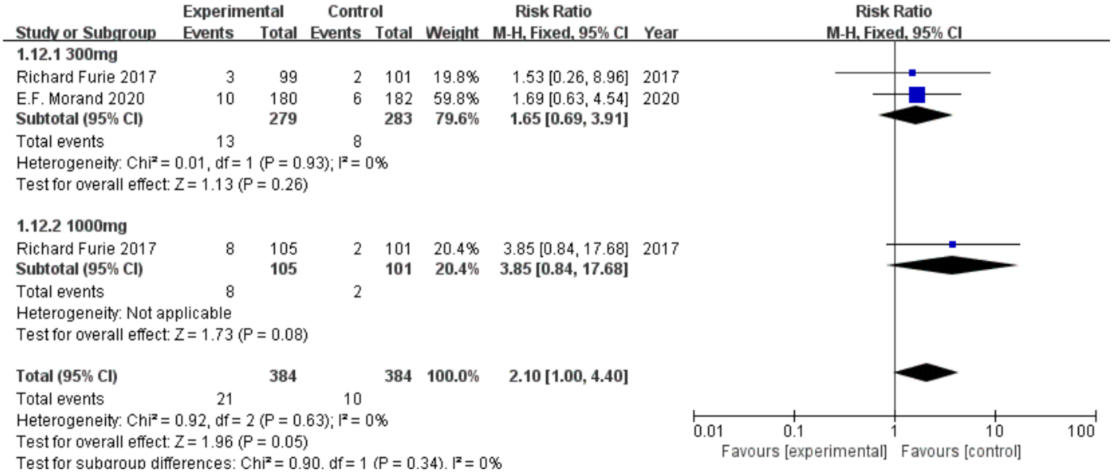

1.13 Tuberculosis

Study or Subgroup	Experimental Events	Experimental Total	Control Events	Control Total	Weight	Risk Ratio M-H, Fixed, 95% CI	Year
**1.13.1 150mg**							
Richard A. Furie 2019	0	93	1	184	32.8%	0.66 [0.03, 15.95]	2019
Ian N Bruce 2021	0	14	0	9		Not estimable	2021
**Subtotal (95% CI)**		**107**		**193**	**32.8%**	**0.66 [0.03, 15.95]**	
Total events	0		1				
Heterogeneity: Not applicable							
Test for overall effect: Z = 0.26 (P = 0.80)							
**1.13.2 300mg**							
Richard A. Furie 2019	1	180	1	184	32.1%	1.02 [0.06, 16.22]	2019
E.F. Morand 2020	3	180	0	182	16.1%	7.08 [0.37, 136.04]	2020
Ian N Bruce 2021	1	13	0	9	18.9%	2.14 [0.10, 47.38]	2021
**Subtotal (95% CI)**		**373**		**375**	**67.2%**	**2.79 [0.57, 13.58]**	
Total events	5		1				
Heterogeneity: Chi² = 0.92, df = 2 (P = 0.63); I² = 0%							
Test for overall effect: Z = 1.27 (P = 0.20)							
**1.13.3 1000mg**							
**Subtotal (95% CI)**		**0**		**0**		**Not estimable**	
Total events	0		0				
Heterogeneity: Not applicable							
Test for overall effect: Not applicable							
**Total (95% CI)**		**480**		**568**	**100.0%**	**2.09 [0.54, 8.16]**	
Total events	5		2				
Heterogeneity: Chi² = 1.42, df = 3 (P = 0.70); I² = 0%							
Test for overall effect: Z = 1.06 (P = 0.29)							
Test for subgroup differences: Chi² = 0.64, df = 1 (P = 0.43), I² = 0%							

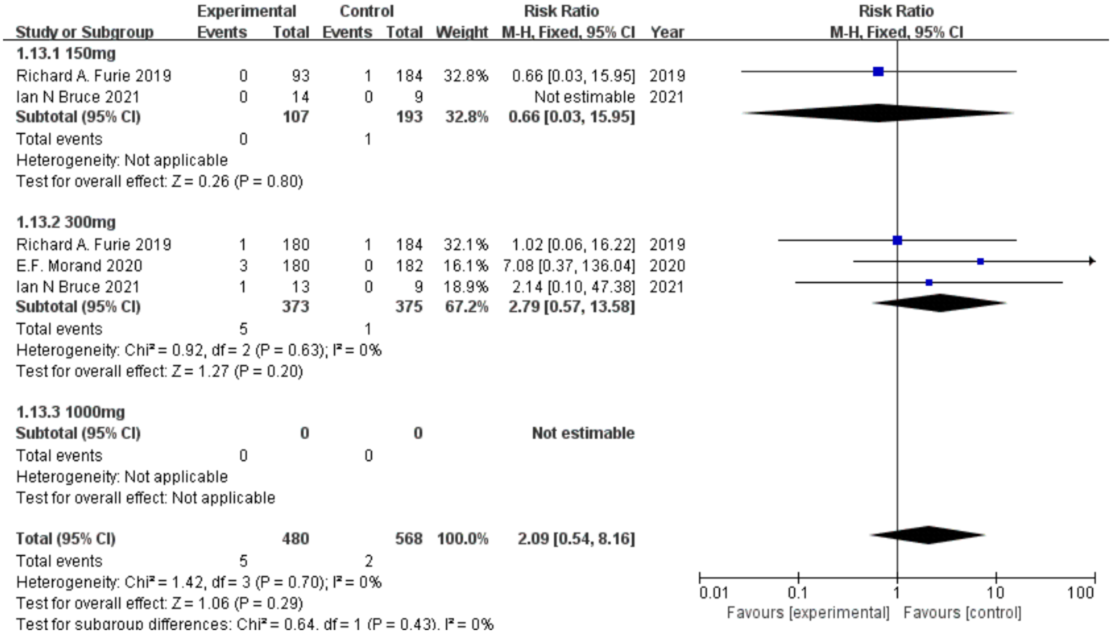

1.14 Malignancy

Study or Subgroup	Experimental Events	Experimental Total	Control Events	Control Total	Weight	Risk Ratio M-H, Fixed, 95% CI	Year
**1.14.1 150mg**							
Richard A. Furie 2019	1	93	1	184	21.3%	1.98 [0.13, 31.28]	2019
Ian N Bruce 2021	0	14	0	9		Not estimable	2021
**Subtotal (95% CI)**		**107**		**193**	**21.3%**	**1.98 [0.13, 31.28]**	
Total events	1		1				
Heterogeneity: Not applicable							
Test for overall effect: Z = 0.48 (P = 0.63)							
**1.14.2 300mg**							
Richard A. Furie 2019	3	180	1	184	31.4%	3.07 [0.32, 29.21]	2019
E.F. Morand 2020	0	180	1	182	47.3%	0.34 [0.01, 8.22]	2020
Ian N Bruce 2021	0	13	0	9		Not estimable	2021
**Subtotal (95% CI)**		**373**		**375**	**78.7%**	**1.43 [0.28, 7.17]**	
Total events	3		2				
Heterogeneity: Chi² = 1.23, df = 1 (P = 0.27); I² = 18%							
Test for overall effect: Z = 0.43 (P = 0.67)							
**Total (95% CI)**		**480**		**568**	**100.0%**	**1.54 [0.38, 6.20]**	
Total events	4		3				
Heterogeneity: Chi² = 1.26, df = 2 (P = 0.53); I² = 0%							
Test for overall effect: Z = 0.61 (P = 0.54)							
Test for subgroup differences: Chi² = 0.04, df = 1 (P = 0.84), I² = 0%							

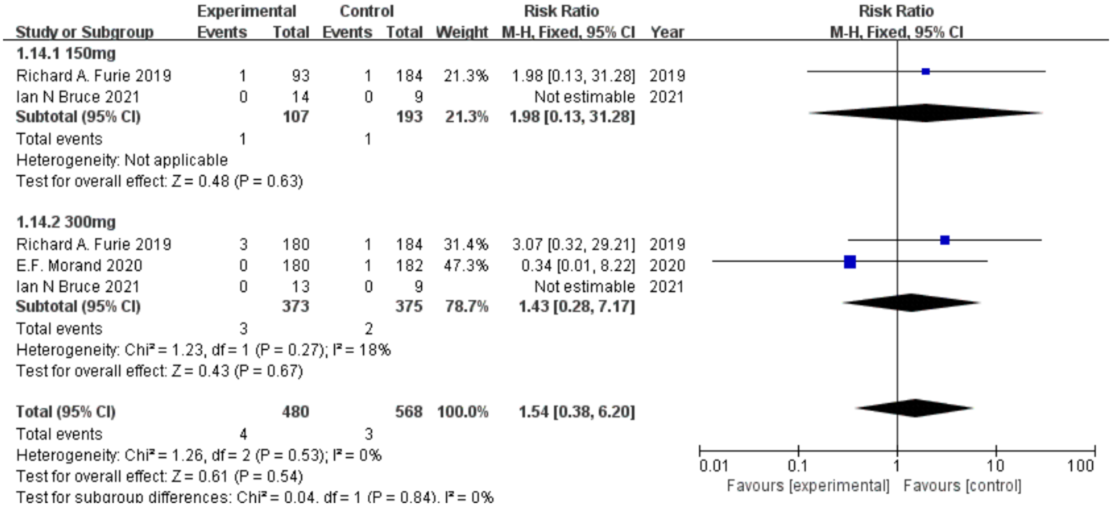

1.15 Infusion-related reaction

Study or Subgroup	Experimental Events	Experimental Total	Control Events	Control Total	Weight	Risk Ratio M-H, Fixed, 95% CI	Year
**1.15.1 150mg**							
Richard A. Furie 2019	9	93	13	184	18.4%	1.37 [0.61, 3.09]	2019
**Subtotal (95% CI)**		**93**		**184**	**18.4%**	**1.37 [0.61, 3.09]**	
Total events	9		13				
Heterogeneity: Not applicable							
Test for overall effect: Z = 0.76 (P = 0.45)							
**1.15.2 300mg**							
Richard Furie 2017	2	99	6	101	12.5%	0.34 [0.07, 1.64]	2017
Richard A. Furie 2019	16	180	13	184	27.0%	1.26 [0.62, 2.54]	2019
E.F. Morand 2020	25	180	14	182	29.3%	1.81 [0.97, 3.36]	2020
**Subtotal (95% CI)**		**459**		**467**	**68.8%**	**1.32 [0.86, 2.04]**	
Total events	43		33				
Heterogeneity: Chi² = 3.84, df = 2 (P = 0.15); I² = 48%							
Test for overall effect: Z = 1.27 (P = 0.20)							
**1.15.3 1000mg**							
Richard Furie 2017	4	105	6	101	12.9%	0.64 [0.19, 2.21]	2017
**Subtotal (95% CI)**		**105**		**101**	**12.9%**	**0.64 [0.19, 2.21]**	
Total events	4		6				
Heterogeneity: Not applicable							
Test for overall effect: Z = 0.70 (P = 0.48)							
**Total (95% CI)**		**657**		**752**	**100.0%**	**1.24 [0.87, 1.79]**	
Total events	56		52				
Heterogeneity: Chi² = 5.14, df = 4 (P = 0.27); I² = 22%							
Test for overall effect: Z = 1.18 (P = 0.24)							
Test for subgroup differences: Chi² = 1.24, df = 2 (P = 0.54), I² = 0%							

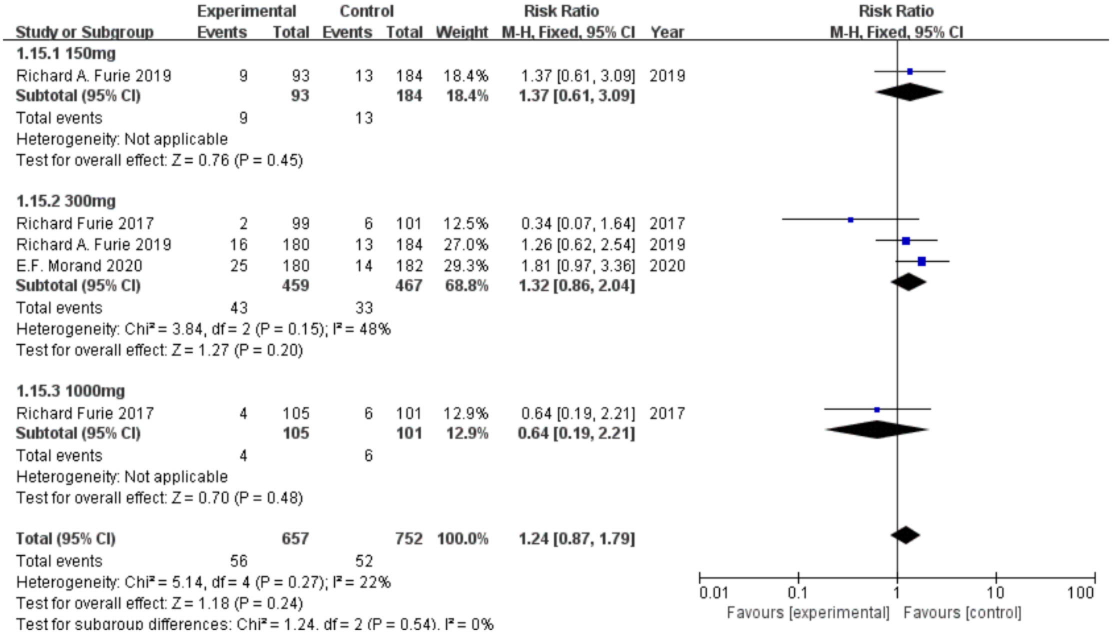

1.16 Anaphylaxis

Study or Subgroup	Experimental Events	Experimental Total	Control Events	Control Total	Weight	Risk Ratio M-H, Fixed, 95% CI	Year
**1.16.1 150mg**							
Richard A. Furie 2019	1	93	0	184	100.0%	5.90 [0.24, 143.54]	2019
Ian N Bruce 2021	0	14	0	9		Not estimable	2021
**Subtotal (95% CI)**		**107**		**193**	**100.0%**	**5.90 [0.24, 143.54]**	
Total events	1		0				
Heterogeneity: Not applicable							
Test for overall effect: Z = 1.09 (P = 0.28)							
**1.16.2 300mg**							
Richard A. Furie 2019	0	180	0	184		Not estimable	2019
Ian N Bruce 2021	0	13	0	9		Not estimable	2021
**Subtotal (95% CI)**		**193**		**193**		**Not estimable**	
Total events	0		0				
Heterogeneity: Not applicable							
Test for overall effect: Not applicable							
**Total (95% CI)**		**300**		**386**	**100.0%**	**5.90 [0.24, 143.54]**	
Total events	1		0				
Heterogeneity: Not applicable							
Test for overall effect: Z = 1.09 (P = 0.28)							
Test for subgroup differences: Not applicable							

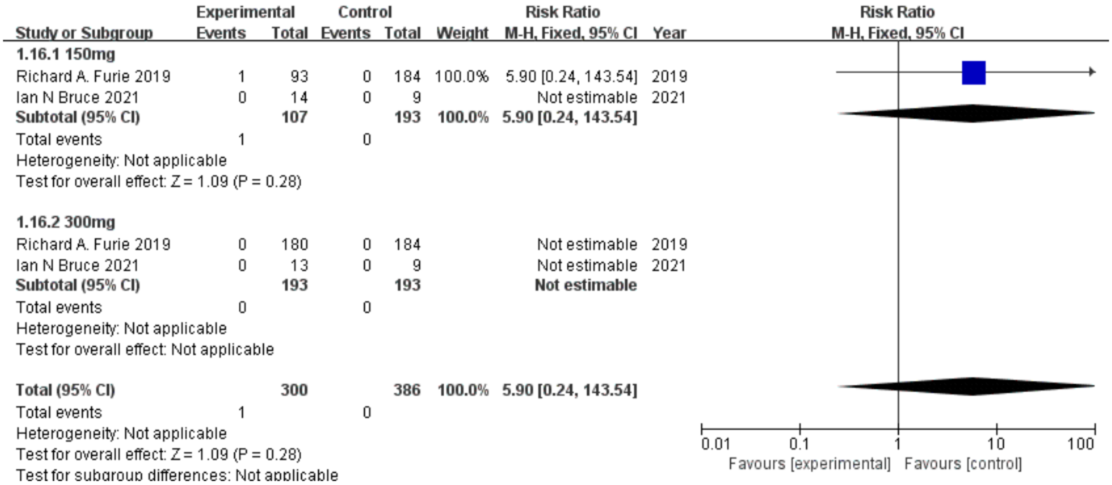

1.17 Non-opportunistic infections

Study or Subgroup	Experimental Events	Experimental Total	Control Events	Control Total	Weight	Risk Ratio M-H, Fixed, 95% CI	Year
**1.17.1 150mg**							
Richard A. Furie 2019	2	93	8	184	23.1%	0.49 [0.11, 2.28]	2019
Ian N Bruce 2021	0	14	0	9		Not estimable	2021
**Subtotal (95% CI)**		**107**		**193**	**23.1%**	**0.49 [0.11, 2.28]**	
Total events	2		8				
Heterogeneity: Not applicable							
Test for overall effect: Z = 0.90 (P = 0.37)							
**1.17.2 300mg**							
Richard A. Furie 2019	9	180	8	184	34.1%	1.15 [0.45, 2.91]	2019
E.F. Morand 2020	5	180	10	182	42.8%	0.51 [0.18, 1.45]	2020
Ian N Bruce 2021	0	13	0	9		Not estimable	2021
**Subtotal (95% CI)**		**373**		**375**	**76.9%**	**0.79 [0.40, 1.57]**	
Total events	14		18				
Heterogeneity: Chi² = 1.32, df = 1 (P = 0.25); I² = 24%							
Test for overall effect: Z = 0.67 (P = 0.50)							
**Total (95% CI)**		**480**		**568**	**100.0%**	**0.72 [0.39, 1.35]**	
Total events	16		26				
Heterogeneity: Chi² = 1.64, df = 2 (P = 0.44); I² = 0%							
Test for overall effect: Z = 1.02 (P = 0.31)							
Test for subgroup differences: Chi² = 0.30, df = 1 (P = 0.58), I² = 0%							

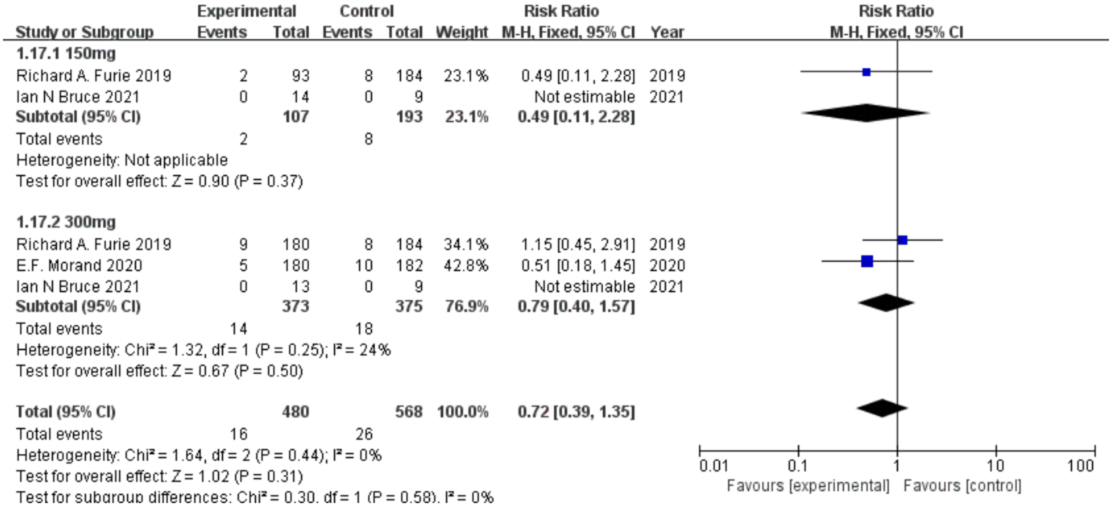

1.18 Opportunistic infections

Study or Subgroup	Experimental Events	Experimental Total	Control Events	Control Total	Weight	Risk Ratio M-H, Fixed, 95% CI	Year
**1.18.1 150mg**							
Richard A. Furie 2019	0	93	1	184	50.5%	0.66 [0.03, 15.95]	2019
Ian N Bruce 2021	0	14	0	9		Not estimable	2021
**Subtotal (95% CI)**		**107**		**193**	**50.5%**	**0.66 [0.03, 15.95]**	
Total events	0		1				
Heterogeneity: Not applicable							
Test for overall effect: Z = 0.26 (P = 0.80)							
**1.18.2 300mg**							
Richard A. Furie 2019	1	180	1	184	49.5%	1.02 [0.06, 16.22]	2019
Ian N Bruce 2021	0	13	0	9		Not estimable	2021
**Subtotal (95% CI)**		**193**		**193**	**49.5%**	**1.02 [0.06, 16.22]**	
Total events	1		1				
Heterogeneity: Not applicable							
Test for overall effect: Z = 0.02 (P = 0.99)							
**Total (95% CI)**		**300**		**386**	**100.0%**	**0.84 [0.10, 6.69]**	
Total events	1		2				
Heterogeneity: Chi² = 0.04, df = 1 (P = 0.84); I² = 0%							
Test for overall effect: Z = 0.17 (P = 0.87)							
Test for subgroup differences: Chi² = 0.04, df = 1 (P = 0.84), I² = 0%							

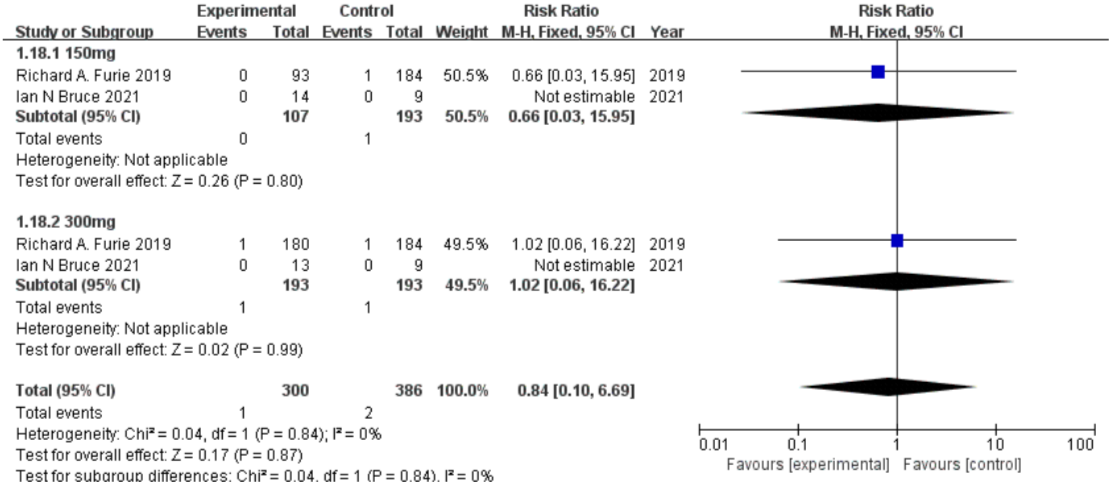

Supplement: Supplementary Figure 1 — Forest plot comparing adverse events of anifrolumab groups and the controls. “Experimental” refers to patients with SLE who received anifrolumab (150mg/300mg/1000mg), and “Control” refers to patients with SLE who received placebo. SLE, systemic lupus erythematosus;95% CI: 95% confidence interval. [file Image_1.pdf]
